# The silicon regulates microbiome diversity and plant defenses during cold stress in *Glycine max* L.

**DOI:** 10.3389/fpls.2023.1280251

**Published:** 2024-01-10

**Authors:** Waqar Ahmad, Lauryn Coffman, Aruna D Weerasooriya, Kerri Crawford, Abdul Latif Khan

**Affiliations:** ^1^ Department of Engineering Technology, Cullen College of Engineering, University of Houston, Sugar Land, TX, United States; ^2^ Department of Biology and Biochemistry, College of Natural Science & Mathematics, University of Houston, Houston, TX, United States; ^3^ Cooperative Agricultural Research Center, College of Agriculture & Human Sciences, Prairie View A&M University, Prairie View, TX, United States

**Keywords:** microbiome, diversity, cold stress, silicon, enzymes, gene expression

## Abstract

**Introduction:**

With climate change, frequent exposure of bioenergy and food crops, specifically soybean (*Glycine max* L.), to low-temperature episodes is a major obstacle in maintaining sustainable plant growth at early growth stages. Silicon (Si) is a quasi-essential nutrient that can help to improve stress tolerance; however, how Si and a combination of cold stress episodes influence plant growth, plant physiology, and microbiome diversity has yet to be fully discovered.

**Methods:**

The soybean plants were exposed to cold stress (8-10°C) with or without applying Si, and the different plant organs (shoot and root) and rhizospheric soil were subjected to microbiome analysis. The plant growth, physiology, and gene expression analysis of plant defenses during stress and Si were investigated.

**Results and discussion:**

We showed that cold stress significantly retarded soybean plants’ growth and biomass, whereas, Si-treated plants showed ameliorated negative impacts on plant growth at early seedling stages. The beneficial effects of Si were also evident from significantly reduced antioxidant activities – suggesting lower cold-induced oxidative stress. Interestingly, Si also downregulated critical genes of the abscisic acid pathway and osmotic regulation (*9-cis-epoxy carotenoid dioxygenase* and *dehydration-responsive element binding protein*) during cold stress. Si positively influenced alpha and beta diversities of bacterial and fungal microbiomes with or without cold stress. Results showed significant variation in microbiome composition in the rhizosphere (root and soil) and phyllosphere (shoot) in Si-treated plants with or without cold stress exposures. Among microbiome phyla, *Proteobacteria*, *Bacteroidota*, and *Ascomycota* were significantly more abundant in Si treatments in cold stress than in control conditions. For the core microbiome, we identified 179 taxa, including 88 unique bacterial genera in which *Edaphobacter*, *Haliangium*, and *Streptomyces* were highly abundant. Enhanced extracellular enzyme activities in the cold and Si+cold treatments, specifically phosphatase and glucosidases, also reflected the microbiome abundance. In conclusion, this work elucidates cold-mediated changes in microbiome diversity and plant growth, including the positive impact Si can have on cold tolerance at early soybean growth stages – a step toward understanding crop productivity and stress tolerance.

## Introduction

Irregular waves of high or low temperatures caused by the recent climate changes drastically hinder the productivity and growth of economically important food crops (Ahmad and Prasad, 2011; Chaudhry and Sidhu, 2022). It has been estimated that an increase of 2°C to 3°C will cause a reduction in plant yield by up to 35% by the end of the 21st century. According to the National Oceanic and Atmospheric Administration, more than $145 billion of economic losses occur due to the impacts of climate change. Some significant crops, such as maize and wheat, will lose their productivity by 24% in the coming decades (Hassanein et al., 2021; Watts et al., 2021). Recently, there have been higher incidences of abnormal freezes or cold waves (2-10°C). Specifically, extremely low night temperatures drastically impact plant growth by causing anatomical, morphological, and physiological changes affecting cell division, photosynthesis, water transport, root architecture, phytohormonal signaling, nutrient uptake, and oxidative stress (Chourasia et al., 2022; Garcia Mendez et al., 2023). Plants mitigate adverse impacts by activating their defense machinery, such as the production of beneficial metabolites and osmolytes, reducing cellular metabolic activities, increasing phytohormones (such as abscisic acid), and producing antioxidants to minimize cellular injuries from reactive oxygen species (ROS) (Waqas et al., 2012; Shaffique et al., 2022).

Silicon (Si) plays a crucial role in plant growth and stress tolerance to minimize the low-temperature impacts. Si – the second most abundant and multi-functional quasi-essential soil element in the earth’s crust is transported by plants in the form of silicic acid (H_4_SiO_4_) (Kim et al., 2016). It ranges from 3-40 mg L^−1^ in soil solution (Ma and Yamaji, 2006; Kim et al., 2016). Chemical or biological weathering processes mobilize Si to form silicic acid in a plant’s rhizosphere (below-ground), convert it into secondary minerals, and adsorb on reactive soil particles (De Tombeur et al., 2021). The plant root uptake it through active or passive transport and assimilate in different organs by forming double silica-cuticle layers in intercellular spaces – enhancing cell wall rigidity through biosilicification, resulting in phytoliths (Ahire et al., 2021). In the last two to three decades, extensive research has been done on how exogenous Si application promotes plant growth, nutrient balance, photosynthesis, and cell redox potential. Furthermore, Si uptake in plants increases stem and leaf rigidity by thickening cell walls and silica cells. This helps plants tolerate stress and avoid dehydration (Khan et al., 2020; Bhardwaj et al., 2022; Bilal et al., 2022; Khan et al., 2022b). Recently, Si has also been found to counteract the negative impacts of heat stress; however, little is known about Si’s role in improving plant defenses during cold stresses.

Microbes, on the other hand, have been known symbionts of plant and playing an essential role in nutrient mobilization in rhizosphere that impacts phyllosphere (above ground) and plant’s health and defenses (Khan et al., 2016). The plant microbiome (assembly of bacterial and fungal species) triggers the plant’s innate immune and defense system, as well as produces a higher amount of extra- and intra-cellular enzymes (cellulase, phosphatases, glucosidases etc) and beneficial metabolites (phytohormones and organic acids), which support the mobilization and transport process of essential nutrients through bioweathering processes (Porras-Alfaro and Bayman, 2011; Chialva et al., 2021; Sachdev and Ansari, 2022). There is an ample literature available on the individual role of microbes in mineral solubilization *in vitro*, however, how microbiome diversity interact with exogenous Si and temperature stress tolerance has not been fully known. Although, studies have shown that taxa from a single genera or family in the rhizosphere or phyllosphere of rice and Arabidopsis plants offer increased stress tolerance (Finkel et al., 2020). Single species of bacterial endophytes (microbes living inside plant tissues) are reported for the accumulation of cold stress-linked metabolites such as essential sugars (starch), amino acids (proline), and phenolic (catechol) compounds in plant tissues (Ayilara et al., 2022). Furthermore, microbiome-mediated temperature tolerance has been reported for maize (Tiziani et al., 2022), rice (Liu et al., 2023), wheat (Chen et al., 2022), and Arabidopsis (He et al., 2022). Microbial communities help soybeans solubilize Si P and produce phytohormones and organic acid (Kang et al., 2017). Specifically, the rhizosphere microbiome can help regulate root architecture and its exudation responses to environmental stimuli. Extracellular enzymes produced by the microbiome have been critical players in macromolecule management and reshaping microbiome diversity and function (Liu et al., 2019). Studies have shown that intrinsic changes in soil physio-chemical properties can reshape microbiome diversity and shift from bulk soil into the root system (Adam et al., 2018). However, the microbiome community diversity is variable across different plants specifically C3 vs C4 plants, which is vastly unknown during stress conditions.

Soybean (*Glycine max* L.) – a C3 plant, has tremendous economic and cultural importance to several countries. Soybeans have been used in various food products, such as soy milk, bean paste, soy sauce, and soybean oil (Khan et al., 2011; Sugiyama, 2019). This is an addition to its use as a bioenergy crop and animal feed. However, soybean plant production is susceptible to low temperatures at early growth stages. Recent studies showed that cold can reduce plant growth by 14% (Kidokoro et al., 2015). Although studies have been conducted on understanding the physiology and genomics of soybeans in temperature tolerance, the plant microbiome interaction has been frequently overlooked as an aspect of elucidating stress tolerance mechanisms. More work must be done on low-temperature stress and its impact on microbiome diversity. Hence, in the current study, we aimed to understand how Si regulates the plant defenses of soybeans in cold stress and how it influences the microbiome diversity and core-microbiome structure in the rhizosphere and phyllosphere compartments of the plant during stress. For this purpose, we have grown the soybean in the presence and absence of cold stress with and without Si additions to assess its influence on plant growth, physiology, cold stress-related gene expression, microbiome structure (bacteria and fungi in soils, roots, and shoots) and activities (extracellular enzymes).

## Materials and methods

### Plant growth conditions and stress treatment

*Glycine max* L. (Fiskeby V Soybean) was acquired from the US Department of Agriculture (Germplasm Resources Information Network (GRIN). Fiskeby V is a hydration and chilling-tolerant variety (Kuczyński et al., 2020). Initially, the soybean seeds were surface sterilized using 2% hypochlorite and 70% ethanol for 2 min each, then washed three times with autoclaved distilled water. The seeds were placed in sterile Petri dishes containing sterile filter paper, and 3-4 ml of autoclaved distilled water was applied to each Petri plate. The seeds were maintained at 25°C ±2°C in complete darkness for four days. The germinated seeds were transferred to pots containing a soil mixture of peat moss (Miracle-Grow, USA), organic topsoil, and Ferti-Lome perlite in 40:30:10 ratios, respectively. Before seed transfer, the soil mixture was autoclaved at 15 Psi, 121°C for 30 min. This is a known method to use a sterilized soil system to assist the plant in establishing its native microbiome during growth stages. This also gives a larger landscape of microbiome development from germination to maturation in the rhizosphere and phyllosphere. Autoclave-based sterilization helps control the natural microbiome in the soil and ensures the assessment of the changes in microbiome diversity (Liu et al., 2022). The plants were grown in a growth chamber (Bioara, MinArc Sys Inc. USA; relative humidity 60%-70%, and light intensity of 800μEm^−2^ s^−1^ (microeinstein of photon flux per square meter per second) from sunlight Z4NW; day/night cycle of 14 h at 28°C and 10 h at 25°C). Initially, the plants were watered with autoclaved DI water. Every alternate day, control plants received 100 ml/pot of only autoclaved DI water to maintain a natural soil moisture level of 50%, and Si-treated plants received 100 ml/pot of 1.0mM Silicic Acid solution (H_2_SiO_4_; Sigma Aldrich, USA) until the completion of the experiment. Thus, before initiating the cold stress, the Si ratio in each pot was approximately 1g/Kg soil. After stage V3 (vegetative stage, 3^rd^ trifoliate), the plants were arranged in a fully factorial experimental design with two explanatory variables: (i) Cold stress and (ii) Silicon (Si) treatments. Thus, the experimental design was comprised of four treatments: (i) control (CT), (ii) Silicon (Si), (iii) Cold stress (CD), and (iv) Silicon+ Cold stress (Si+Cold). At the V3 stage, the cold stress was induced by exposing the plants to 8°C to 10°C for two weeks. The 16 h cold stress exposure was divided into five parts with a gradual decrease from 25°C to 20°C (2 h) to 15°C (2 h) to 5°C (8 h) and increased to 25°C in a similar pattern to avoid sudden shocks. Each treatment was comprised of 21 plants. After two weeks of treatments, plant growth parameters were measured. The plant and soil samples were harvested using liquid nitrogen and kept at –80°C until further analysis.

### Plant growth parameters and oxidative stress analysis

Plant growth attributes such as shoot length, root length, and biomass were noted. Chlorophyll content was recorded using a chlorophyll meter. Oxidative stress enzymes catalase (CAT), superoxide dismutase (SOD), peroxidase (POD), and polyphenol oxidase (PPO) were analyzed for the phyllosphere and rhizosphere of all treatments. Shoot and root samples (100 mg) were ground using a chilled mortar and pestle, then combined with extraction buffer (1 mM Tris-HCl + 6 mM MgCl_2 + _1 mM EDTA + 3.5 PVP) to homogeneity, followed by centrifugation (4,000 rpm for 10 min) in a refrigerated centrifuge. To quantify POD, extract (100 μL) was combined with sodium phosphate buffer (0.1 M, pH 6.8), H_2_O_2_ (50 μL of 50 µM), and pyrogallol (50 μL of 50 µM) and incubated at room temperature for 5 min, followed by the addition of H_2_SO_4_ (5% v/v). The absorbance was measured at 420 nm. The exact wavelength of measurement (420 nm) and a similar reaction mixture composition earlier used for POD were used to quantify PPO without H_2_O_2_ (50 µM). The CAT activity was assayed as described by Aebi (1984). Briefly, the crude enzyme extract was added to H_2_O_2_ (0.2 M) in phosphate buffer (10 mM, pH 7.0), and the CAT activity was determined as the decrease in absorbance at 240 nm and expressed as units (1 U of CAT was defined as μg H_2_O_2_ released mg protein^–1^ min^–1^). According to (Giannopolitis and Ries, 1977), SOD was assayed based on nitro blue tetrazolium (NBT) reduction by measuring the absorbance at 560 nm (1 U of SOD was defined as the enzyme amount caused 50% NBT reduction inhibition). The absorbance was measured using TECAN Spark 10M spectrophotometer.

### Gene expression analysis

Following the manufacturer’s protocol, RNA was extracted from all the treatments using the GeneJET PCR Purification Kit (Thermo Scientific). RNA quality and quantity were analyzed using Qubit 4.0 (Qubit RNA IQ Assay and RNA HS Assay kits) and gel electrophoresis. All the RNA samples were normalized to 100 ng/μL. A High-Capacity cDNA Reverse Transcription kit (Applied Biosystems) was used for cDNA synthesis as per the provided protocol using a PCR thermo-cycler (25°C for 10 min, 37°C for 2 hours, and 85°C for 5 min). qRT-PCR was performed using the QuantStudio 7 Flex system (Applied Biosystems). The PCR reaction was carried out in a total volume of 20 μL reaction mixture containing 10 μL master mix (PowerUp™ SYBR™ Green Master Mix),1 μL primer, 7 μL RNase free water, and 1 μL cDNA, with each reaction repeated three times. The selected gene primers were designed using the primer three program and are provided in **Table S1**. Actin (housekeeping gene) was used to normalize all gene expression and the expression level in control plants compared with Si, cold and Si+cold treated plants was calculated using the comparative ΔΔCt method.

### Extracellular enzyme analysis

We used the previous protocol, Marx et al. (2005) and Jian et al. (2016) for extracellular enzymes. A stock solution of MUB (4-methylumbelliferone, ten mM) was prepared in methanol and diluted to 1 µM in sodium acetate (pH 5.2) buffer. The rhizospheric soil samples from all treatments were incubated in sodium acetate buffer (pH 5.2) for 24 h on shaking (150 rpm), and the supernatants were harvested using centrifugation (4°C, 12,000 rpm for 20 min). The filtrates were syringe filtered (0.22 μm) to remove traces of turbidity. The exozymes β-D-cellubiosidase (BDC), α-Glucosidase (AG), β-Glucosidase (BG), N-acetyl-β-Glucosaminidase (NAG), and Phosphatase (Phos) were quantified on a fluorescence spectrophotometer. For each type of enzyme analysis, a minimum of five replicates for each substrate (F + buffer + substrate), a quenched standard (sample + buffer + 4-MUB), and a substrate control (pad + substrate) were maintained. The pre-optimized fluorescence spectrophotometer (Shimadzu, Tokyo, Japan) read the absorbance with 360nm excitation and 460nm emission at time zero and 30-min intervals for 2 hours (Stroud et al., 2022).

### Microbiome DNA extraction

DNA was extracted from leaves, roots, and rhizospheric soil from all four treatments. The MagMAX™ Plant DNA Kit (Thermo Scientific) was used for leaves and roots samples, whereas the ZymoBIOMICS™ DNA Miniprep kit (Zymo Research) was used for rhizospheric soil samples. For the endospheric microbiome of root and shoot parts, we followed the method of Khan et al. (2022a) to perform surface sterilization. The DNA extraction was performed according to the manufacturer’s protocols. The final DNA pellets were eluted in 60 μL of elution buffer (included in the kit). The quantity and quality of extracted DNA samples were analyzed using a Thermo Scientific NanoDrop Lite Spectrophotometer and Invitrogen™ Qubit™ 4.0 Fluorometer and visualized using gel electrophoresis.

### Metagenome sequencing

Amplicon sequencing was performed on all DNA samples. PCR-free libraries were generated by amplifying 16S rRNA (V5-V7) and internal transcribed spacer (ITS1-5F for leaf and root and ITS-5F for soil) regions for bacterial and fungal communities, respectively. Peptide nucleic acid clamps were utilized to minimize chloroplast and mitochondrial DNA contamination. An Illumina MiSeq instrument (Illumina Inc., San Diego, CA, USA) operating with v2 chemistry (User Guide Part # 15,027,617 Rev. L) was used using a paired-end sequencing approach of 300 bp. All quality reads included in the present study are submitted to NCBI under Bioproject PRJNA981149 and are available under the SRR24887345-SRR24887340 (ITS), SRR24880681- SRR24880652 (16S).

### Bioinformatics analysis

QIIME 2.0 (Bolyen et al., 2019) was used for analyzing sequencing reads. Reads (**Supplementary Tables S2–S5**) were checked for quality with fast QC. DADA2 was used for denoising and generating the ASVs (amplicon sequence variants, **Supplementary Tables S5, S6**) (Callahan et al., 2017). In the denoising, sequences were filtered, trimmed (low quality), and chimeric sequences were removed (Callahan et al., 2017). The SILVA classifier for 16S and UNITE database for ITS was used for the taxonomic classification (Quast et al., 2012; Nilsson et al., 2019). The mitochondrial and chloroplast sequences were filtered. For alpha and beta diversity, the Shannon diversity index and Bray-Curtis PCoA matrix were generated and exported to Rstudio for visualization and analysis. Permutative multivariate analysis of variance (PERMANOVA, 999 permutations) was used to test the significant effects of factors (treatments and plant parts) and their interaction on fungal and bacterial community structures using the Adonis function. ANCOM-BC2 (Nilsson et al., 2019) was used to test the effects of the factors on the differential abundance of the fungal and bacterial communities. The datasets were divided into cold vs. control and Si vs. control. For the core microbiome analysis, the plants’ three compartments (leaf, root, and soil) were clustered together for each treatment to determine the shared and unique core microbiome ASVs (Khan et al., 2022a).

### Statistical analysis

In the current study, at least three replicates per treatment were analyzed. The data is presented as mean ± standard error. One-way and two-way analyses of variance (ANOVA) were used to determine the significant differences between control vs. cold, Si vs. cold stress, and control. This helped to understand the impact of cold stress on plants and Si during stress. The mean values were considered significant at *p<*0.05 and were calculated by GraphPad Prism Version 9.01 (GraphPad Software, San Diego, CA, USA). The GraphPad was used for the ANOVA analysis.

## Results

### Silicon application improves plant growth and biochemical activities during cold stress

To determine whether silicon (Si) application influenced plant resilience against cold stress, we measured plant growth parameters and biochemical activities. Si positively impacted plant growth (biomass, shoot, and root lengths) with or without exposure to cold stress conditions. Si increased biomass by 11% to 31% (*p<*0.05, with or without cold stress, respectively). A similar trend was observed for the shoot length, which was significantly increased (*p<*0.05), 24 to 34% relative to the control by Si and in combination with cold stress, respectively (**Supplementary Figure S1**). Root length was also positively influenced by the application of Si (*p<*0.05; ~6%), and Si also ameliorated the negative impacts of cold-induced tissue desiccations (~8.7%). Wilting and curling were significantly more pronounced in cold stress without Si application than with the Si application. We noticed nodule formation (~5 per plant) and early flowering in Si application. Thus, results suggest that cold stress delayed the flowering and nodule formation in soybean plants compared to control conditions (**Supplementary Figure S1**).

**Figure 1:**
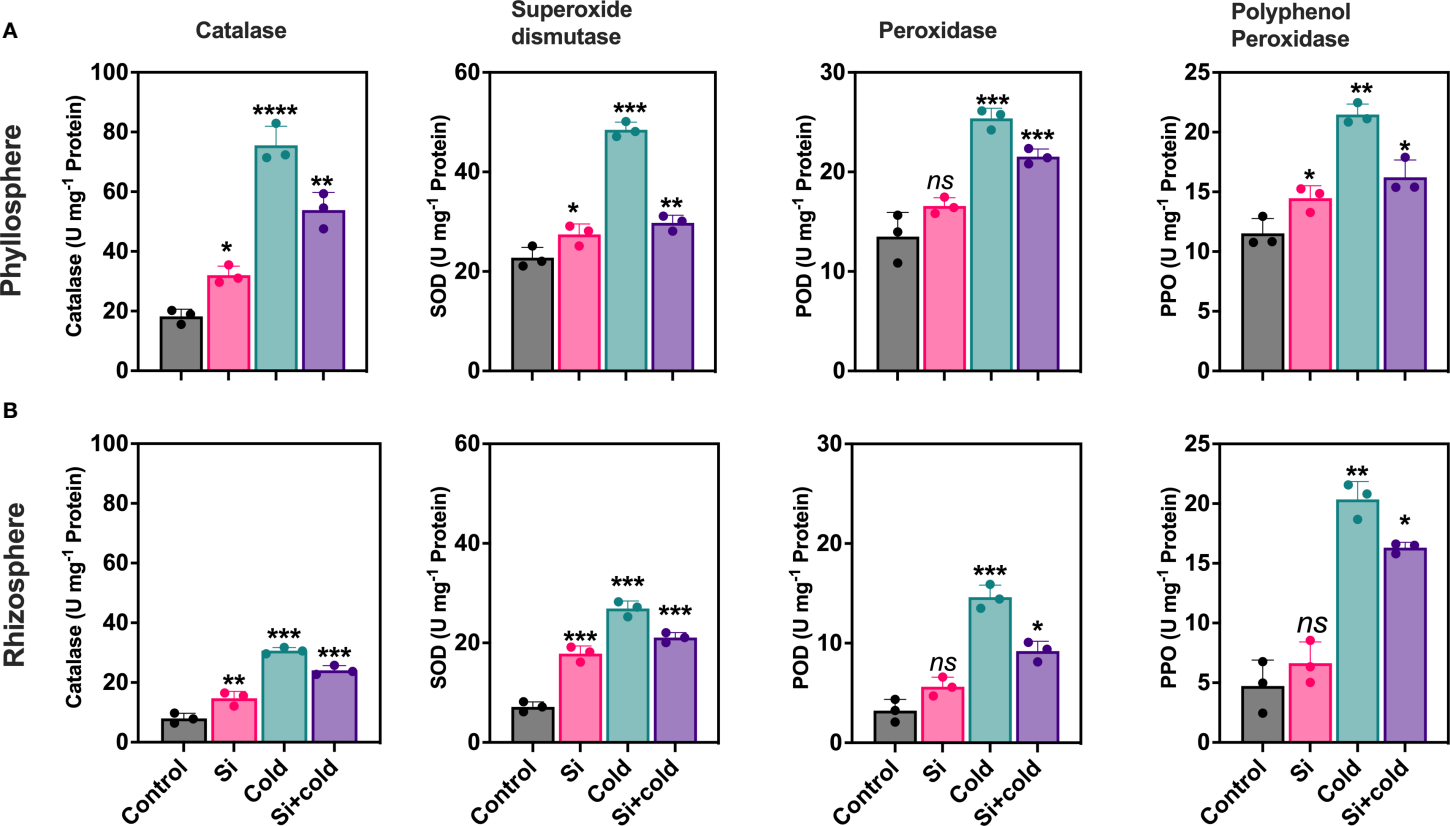
Impact of exogenous silicon supplementation on antioxidant enzyme activities in control and cold stress conditions. **(A)** Phyllosphere **(B)** Rhizophere.The bars showing *, **, ***, and **** are significantly different compared to the control as analyzed by one-way ANOVA analysis. ‘ns’ shows a nonsignificant difference compared to the control.

Cold stress preferentially modulated oxidative stress-induced enzymatic activities (catalase – CAT, superoxide dismutase – SOD, peroxidase – POD, and polyphenol oxidase - PPO). Results showed that the antioxidant enzymes were significantly variable across root-to-shoot parts of the plant during cold stress and, importantly, that Si treatment reduced antioxidant enzyme activities in cold-treated plants. Control plants had the lowest levels of CAT activity in roots and shoots. Contrarily, the cold stress-treated plants increased shoot CAT activity 4.1-fold and root CAT activity two-fold. The application of Si decreased CAT activities (29%; *p<*0.05) during cold stress conditions (**Figure 1**). The shoot part had two-fold higher CAT activities than the root parts across all treatments. SOD activities were significantly higher in cold stress treatment in roots (3.7-fold; *p<*0.05) and shoots (2.1-fold; *p<*0.05) compared to control plants.

Interestingly, Si exponentially increased the CAT activities (*p<*0.05; 2-folds) in the shoot. Contrarily, the Si application in the cold significantly reduced the SOD activities in both shoot and root parts (Si+cold) compared to the control. However, this level was still comparatively higher than control plants with or without cold stress. In the case of PPO, the root-to-shoot enzyme activities were not significantly different in overall content. However, a similar pattern of reduced activities was noticed in Si treatments than in control plants with or without cold stress. A similar trend in enzymatic regulation was also noticed for POD across root-to-shoot components and treatments (Si with or without cold stress; **Figure 1**).

### Regulation in gene expression pattern in Si and cold stress treatments

**Figure 2:**
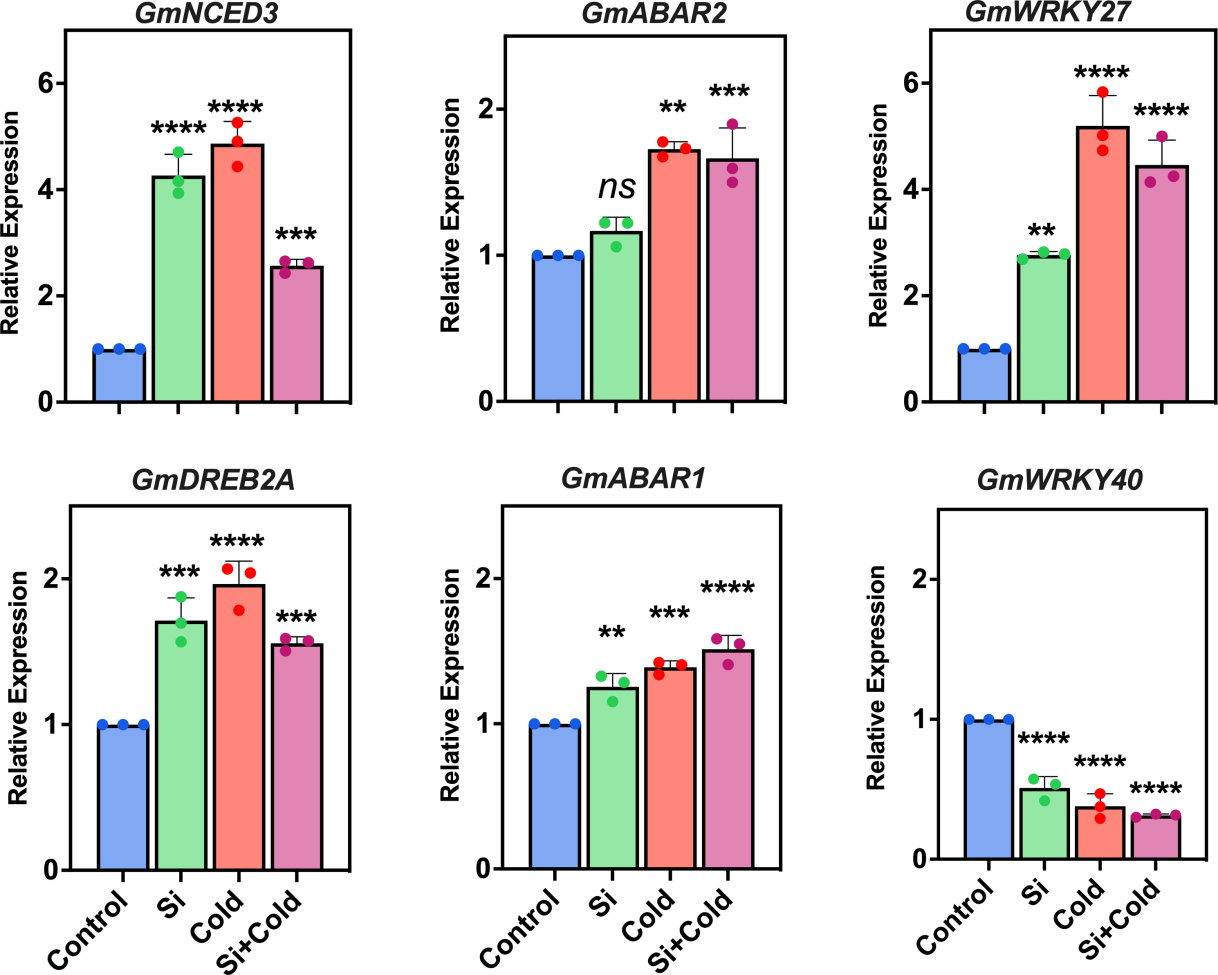
Molecular expression of osmoregulatory and ABA biosynthesis-related genes using quantitative real-time PCR (qRT-PCR) in cold stress and control conditions supplemented with and without exogenous Si. The values represent the mean values of three replicates and show the standard deviation of relative expression. The bars showing **, *** and **** are significantly different in their expression levels compared to the control as analyzed by one-way ANOVA analysis. ‘ns’ shows nonsignificant expression levels compared to the control.

Since environmental cues can stimulate large-scale gene expression patterns, specifically stress-responsive defense mechanisms, we assessed the mRNA expression patterns of stress signaling and dehydration-related genes (**Figure 2**). Here, we analyzed the gene expression of the shoot part as the dehydration was significantly pronounced. We also performed a similar analysis for the root part, which either showed poor gene expression profiles or were undetected. Overall, the results revealed that cold stress significantly upregulated the signaling and expression of most of the analyzed genes. Si treatment did not always mitigate the impact of cold stress on gene expression. In the case of *9-cis-epoxy carotenoid dioxygenase* (*GmNCED3*), the expression was significantly (F1,48 = 6.2; *p<*0.05) increased by cold stress treatment up to 4.8-fold, where the application of Si and combination with cold stress, the relative expression were 4.2-fold and 2.5-fold, respectively, compared to the control treatment (**Figure 2**). Similarly, the *dehydration-responsive element binding protein (GmDREB2A)* was also significantly expressed (*p<*0.05) in all treatments compared to the control. We observed that cold stress treatment has significantly (*p<*0.05) increased the mRNA gene expression pattern of *GmDREB2A* three-fold. In contrast, the Si application modulated this to lower levels (1.9 fold decrease) – suggesting a reduced dehydration impact by cold stress conditions. However, this was still significantly higher in control plants. Interestingly, the *GmNCED3* gene expression levels were significantly higher than the *GmDREB2A* – suggesting a more potent role in cold stress.

Since the abscisic acid (ABA) related pathway is crucial in stress tolerance, we assessed the gene expression of ABA receptor-like protein *ABAR1* and *ABAR2* genes. *ABAR1* gene expression was significantly (*p<*0.01) higher across all treatments (1.2-fold Si, 1.3-fold cold, and 1.5-fold Si+cold) than in control. Contrarily, the *ABAR2* gene was highly significant (*p<*0.05) in its expression pattern during cold stress. In contrast, Si reduced the expression patterns by 0.4-fold in cold stress (Si+cold treatment) compared to cold stress plants (cold treatment). However, the Si vs. non-Si control plants did not differ in their expression of the *ABAR2* gene. In the case of the most common and largest transcription factors and stress-responsive gene families - the WRKY protein domain - we assessed the two most known genes, *GmWRKY40* and *GmWRKY27*. The expression of the *GmWRKY27* gene was significantly higher (*p<*0.05) in all treatments compared to the control. Interestingly, the relative expression of *GmWRKY27* was highest in cold stress, with a 4-fold increase compared to the control. In contrast, it was significantly reduced (1.76-fold) in Si application during cold stress. Surprisingly, the relative gene expression of *GmWRKY40* was exponentially lower in all treatments compared to the control – suggesting a lack of function during cold or Si treatments (**Figure 2**).

### Microbiome composition in cold stress

**Figure 3:**
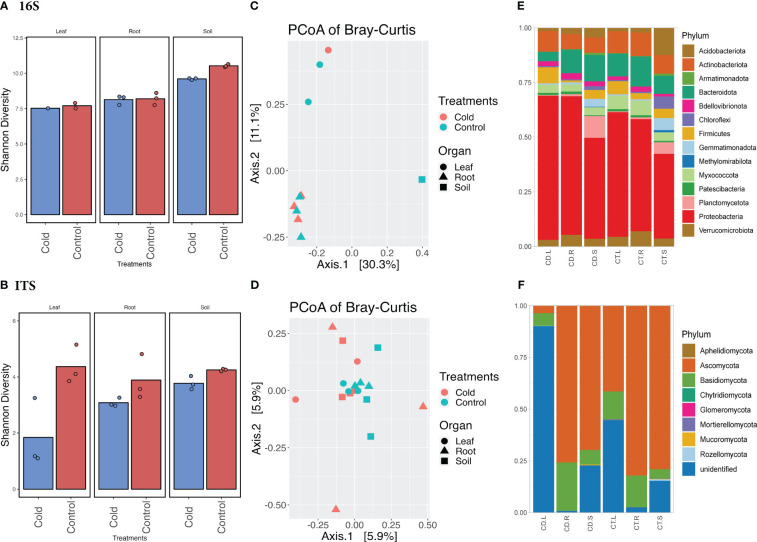
Microbiome diversity and phyla abundance in the cold vs. control treatments. **(A)** Shannon diversity index of bacterial biome in the cold vs. control treatments. **(B)** Shannon diversity index of fungal biome in the cold vs. control treatments. **(C)** Principal coordinates analysis (PCoA) based on Bray-Curtis distances of bacterial biome in the cold vs. control treatments. **(D)** Principal coordinates analysis (PCoA) based on Bray-Curtis distances of fungal biome in the cold vs. control treatments. **(E)** Comparison of the phylum-level distribution of the bacterial microbiota between cold stress and control treatments. **(F)** Comparison of the phylum-level distribution of the fungal microbiota between cold stress and control treatments. CD.L, CD.R, and CD.S are cold-treated leaf, root, and Soil, respectively, whereas CT.L, CT.R, and CT.S are control leaf, root, and soil, respectively.

Our results from plant growth, enzymatic activities, and related gene expression profiles showed that cold stress drastically impacts soybean plant growth. To further elucidate how the aboveground and belowground microbiome diversity is regulated by Si with a combination of cold stress treatment, we performed an in-depth analysis of bacterial and fungal communities. For this purpose, we assessed the microbiome richness and abundance in the soybean plants’ rhizosphere (soil and root) and phyllosphere (leaf). The results showed significant variation (*p<*0.05) in microbial diversity in leaf and root compartments during cold stress (**Figure 3**). This suggests that cold stress has significantly influenced the endospheric microbiome rather than the soil microbiome. The change in Shannon and Bray-Curtis diversity can be observed in shifts in community composition. A two-way ANOVA multiple comparisons of the Shannon diversity dataset (**Supplementary Table S7**) for bacterial and fungal communities showed that the diversity was significant (*p<*0.05) in bacterial communities during cold stress compared to the control. Overall, the Shannon diversity was highly significant (*p<*0.0001) in all three compartments of the plants, whereas it was significant (*p<*0.05) in treatments (cold vs. control). The Shannon diversity in the control soil was higher (10.452) than in cold-treated soil (9.528) for the bacterial microbiome. Although, the Shannon diversity was insignificant (*p<*0.05) in all three plant compartments in cold stress compared to the control for the fungal microbiome. However, the difference in Shannon diversity for fungal communities showed that the highest diversity observed in the leaf (4.376; *n*=3) part in the control condition compared to the cold stress (1.843, *n*=1) (**Figure 3**; **Supplementary Tables S8, S9**).

The principal coordinate analysis (PCoAs) of Bray–Curtis distances and permutational multivariate ANOVA (PERMANOVA) determined bacterial and fungal microbiome dissimilarities. The first coordinate (PCoA1) described 30.3% of the variance, and the second (PCoA2) described 11.1% variance (**Figure 3**) among the cold-stressed vs. control. The PERMANOVA analysis was not significant for all three compartments of the plants. When analyzing all three compartments in cold stress vs. control individually, the PERMANOVA showed a significant variance of (Pr(>F) = 0.01) in the leaf and root compartments of the plants (**Supplementary Table S10**). Similarly, the PCoAs of Bray–Curtis distances showed 5.9% variance at both coordinates with non-significant PERMANOVA in cold stress vs. control plants for fungal microbiomes (**Figure 3**; **Supplementary Table S10**).

### Abundance of the taxon in cold stress

**Figure 4:**
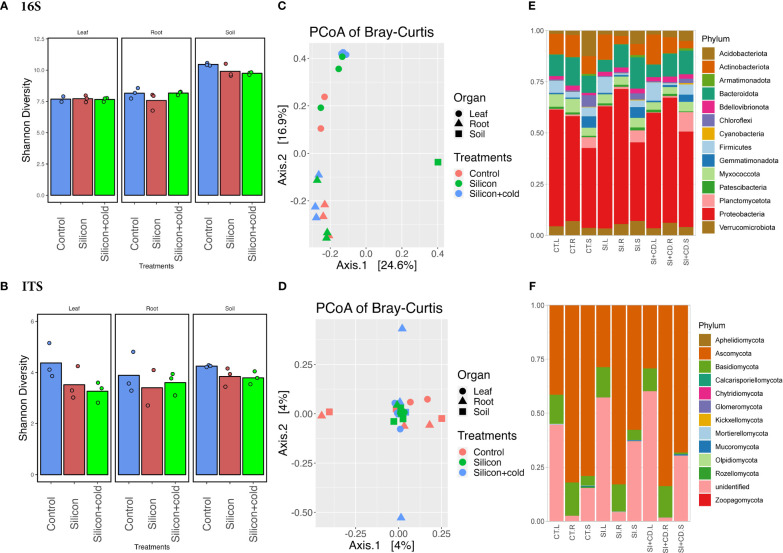
Microbiome diversity and phyla abundance in Si vs. control treatments. **(A)** Shannon diversity index of bacterial biome in Si vs. control treatments. **(B)** Shannon diversity index of fungal biome in the cold vs. control treatments. **(C)** Principal coordinates analysis (PCoA) based on Bray-Curtis distances of bacterial biome in Si vs. control treatments. **(D)** Principal coordinates analysis (PCoA) based on Bray-Curtis distances of fungal biome in the cold vs. control treatments. **(E)** Comparison of the phylum-level distribution of the bacterial microbiota between Si, Si+Cold, and control treatments. **(F)** Comparison of the phylum-level distribution of the fungal microbiota between Si, Si+Cold, and control treatments. CT.L, CT.R, and CT.S are control leaf, root, and soil, respectively, and SI.L, SI.R. SI.S are Si-treated leaf, root, and soil, respectively, whereas SI+CD.L, SI+CD.R, and SI+CD.S are Si+Cold leaf, root and soil respectively.

The bacterial and fungal taxa (phylum and genus) showed variable but interesting diversity across all three compartments (soil, root, and leaf) in cold and control treatments (**Figure 4**). The abundance (number of ASVs after the denoising and filtration) of bacterial taxa shrank in the leaf part in control treatments (47,220= *n*=1, ASVs) compared to the root (226,380 ASVs; *n*=3) and soil (230,280 ASVs; *n*=3) (**Supplementary Table S11**). A similar pattern of abundance was observed in cold treatments for leaf, root, and soil, however, in cold treatments, the abundance of bacterial taxa were reduced in all three parts. Interestingly, the fungal phyla ASVs in the control leaf were much lower (311,682 *n*=3) compared to the cold condition; the cold caused an increase in the leaf fungal phyla (346,611 *n*=3). Although the fungal phyla ASVs were higher in control soil and root than in leaf. A similar ASVs (abundance pattern) was observed in bacterial and fungal genera (**Supplementary Tables S12, S13**).

Moreover, pairwise differential abundance analysis at the ASV level was performed to know the effect of cold stress on the distribution. As per ANCOM-BC2 results, the cold stress vs. control showed 17 bacterial taxa to be differentially abundant in either treatment, among which five genera (*Granulicella*, *WPS-2*, *Parvibaculum*, *RS25G*, and *uncultured_129)* were differentially abundant in cold vs. control treatments. Contrarily, the ANCOM-BC analysis showed no fungal taxa differentially abundant in cold vs. control treatments (**Supplementary Table S14**).

We selected the highly abundant phyla from all the detected taxons for bacterial and fungal microbiomes. Looking deep into the abundances, the *Proteobacteria* was a highly abundant phylum in all three compartments of the plant, both in control (46%, 227,395 ASVs) and cold treatments (52%, 249,412 ASVs), followed by *Bacteroidota*, 11% in both cold stress (54,097 ASVs) and control (54243 ASVs). The abundances were significantly different in leaf, root, and soil. For example, Firmicutes were more abundant phyla in the leaf, whereas *Acidobacteriota* and *Planctomycetota* were abundant in the soil (**Figure 3**). Furthermore, *Ascomycota* was highly abundant (69%, 722,441 ASVs) in control and cold stress (73%, 510,424 ASVs). As shown in **Figure 3**, *Ascomycota* was a more abundant phylum in root and soil compartments of the plants in control and cold treatments. The diversity was lower in fungal than bacterial taxa compared to fungal abundance across the three compartments—the leaf compartments recruited mostly unidentified fungal phyla. *Basidiomycota* was the second most abundant phylum in both cold and control treatments. However, the abundance in cold stress was higher (16%, 110,854 ASVs) than the control (12%, 118,375). The unidentified phyla also had a significant share, 19% in cold stress and 11% in control. Diving deep into genera, the root part was more diverse in fungal abundances, recruiting *Fusarium, Coprinellus, Triangularia*, and *Gibberella* genera (**Supplementary Figures S3**, **S5**). Interestingly these were more abundant in cold treatments as compared to the control. Moreover, based on the number of ASVs, the cold conditions reduced the fungal phyla.

### Silicon changes the structure of microbiome species with or without the cold stress

Although the exogenous Si showed a positive impact on plant growth parameters and significantly reduced some of the gene expression and enzymatic activities related to cold stress, however, we performed microbiome diversity and richness analysis to understand further and elucidate how it changes the microbiome structure and diversity across different treatments. The Shannon diversity was insignificant (*p<*0.05) by two-way ANOVA analysis in Si treatments for both bacterial and fungal microbiomes; however, the bacterial diversity was found to be highly significant (*p<*0.0001) across all three compartments of the plants. The diversity of bacterial communities was significantly higher in the soil than leaf and root. The Shannon diversity was highest in soil for all three treatments (**Figure 4**; **Supplementary Tables S8, S9**). Interestingly, the fungal microbiome’s Shannon diversity was found to be highest (4.376) for the control leaf as compared to the root (3.888) and soil (4.254). In contrast, it was the highest in soil (3.792) compared to the root (3.607) and leaf (3.263) in the Si+Cold treatment (**Figure 4**). According to the principal coordinate analysis (PCoAs) of Bray–Curtis distances and permutational multivariate ANOVA (PERMANOVA), the fungal microbiome diversity was insignificant (*p<*0.05). However, the PCoAs of Bray–Curtis distances showed a 4% variance (PCoA1 and PCoA2) (**Figure 4**; **Supplementary Figure S2**, **Supplementary Table S10**).

### Abundance of the taxon in Si-treatment

We analyzed the bacterial and fungal microbiome’s phylum and genus level abundance for control vs. Si and Si+Cold treatments (**Figure 4**; **Supplementary Figures S3**, **S4**). The highest fungal phyla level ASVs were found in Si-treated soil (379,250) as compared to control (336,506) and Si+Cold soil (332,415) (**Supplementary Table S11**). Similarly, the highest fungal (phylum) ASVs (361,985) were identified in Si+Cold treatment compared to Si (325,266) and control (311,682). The Si-treated soil had 265,046 ASVs, whereas the Si+Cold and control soil had 259,596 and 230,280 ASVs (phylum). Similarly, the case of pairwise differential abundance analysis at the ASV level showed interesting results for Si and control treatments. In Si vs. control and Si+Cold vs. control, 17 taxons were differentially abundant in either treatment compared to control. The *Cellulomonas* genus was found to be true (differential intercept) in all pairwise comparisons across the treatments, whereas *Actinobacteria* was differentially abundant in Si-treated and Si+Cold treatments (**Supplementary Tables S12, S13**).

Bacterial and fungal phyla abundances were analyzed in Si and Si+Cold treatments compared to the control. In fungal phyla, *Ascomycota* was found to be highly abundant in all treatments i.e., 69% (722,441 ASVs) in control, 60% (516,302 ASVs) in Si+Cold, and 54% (637,305 ASVs) in Si-treated plants. Interestingly, unlike the cold vs. control, here the unidentified phyla were highly abundant in Si treatment (36%, 337,234) and Si+Cold treatment (31%, 324,814 ASVs) after the Ascomycota except (**Figure 4**). Similarly, *Rozellomycota* was found to be more abundant in the leaf part, followed by the root, whereas in soil, its abundance was comparatively lower compared to all three treatments. Interestingly, in the soil part, we found *Mucoromycota*, *Zoopagomycota*, and *Mortierellomycota* more abundant than root and leaf (**Figure 4**; **Supplementary Figure S4**).

In genus-level diversity, control and Si+Cold treatments recruited more fungal inter-genera diversity. After the unidentified genera, control and Si+Cold root had high abundances of *Coprinellus*, *Fusarium*, and *Gibberella* genera. Interestingly, *Gibberella* and *Humicola* were more abundant in Si+Cold treatment. Furthermore, in the bacterial microbiome, the highest abundant taxa were *proteobacteria* in all three treatments, i.e., 51% (243,451 ASVs) in Si treatment and 50% (191,197 ASVs) in Si+Cold treatment. Interestingly, the soil part of all the treatments showed higher bacterial phyla abundances than leaf and root (**Figure 4**; **Supplementary Figure S4**). The abundance of *Bacteriodota* was higher in Si-treated soil as compared to Si+Cold soil and control soil. Similarly, *Firmicutes* were highest in the leaf part, especially in the Si+Cold leaf, compared to the control. The root and leaf compartments have diverse genera, whereas soil has mostly uncultured genera. *Noviherbaspirillum, Streptomyces, Bacillus*, and *Bradyrhizobium* were more abundant in root and leaf than in soil. However, *Sphingomonas* and *Burkholderia* were more abundant in the soil than leaf and root (**Supplementary Figures S3**, **S5**).

### Core-microbiome structures of cold and Si treatments

**Figure 5:**
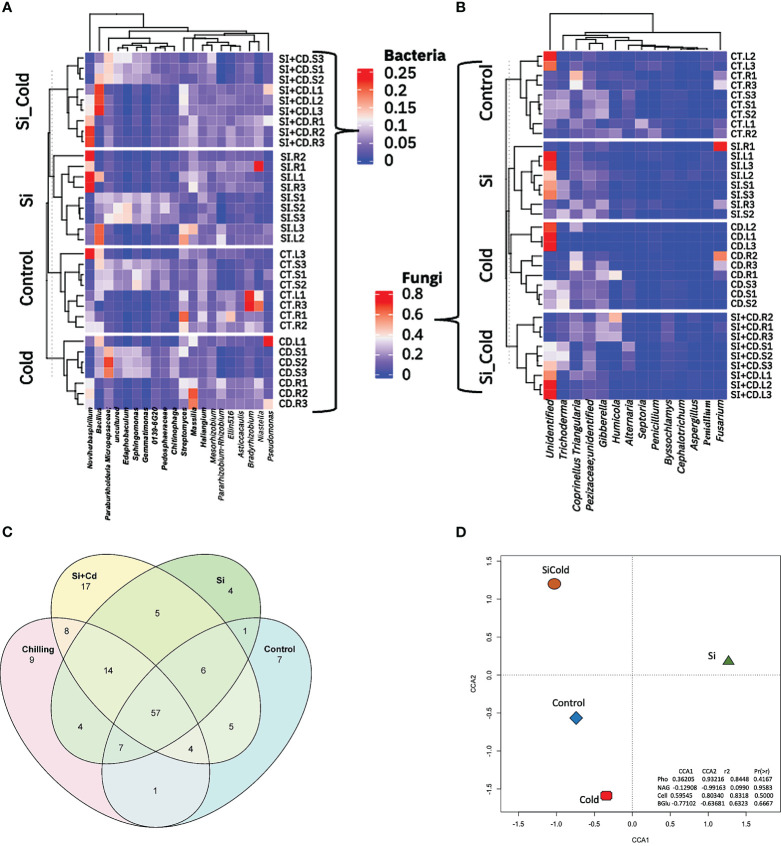
Genera-level microbial abundance with or without the presence of Si and cold stress. **(A)** shows the top 14 bacterial and **(B)** fungal genera found in control, Si, cold and Si+Cold treatments of soybean plants’ leaf, root, and soil parts. **(C)** shows the core microbiome of bacteria and its distribution across different treatment structures. **(D)** The Canonical Correspondence Analysis (CCA) of genera level abundances about treatments and extracellular enzymes of soil.

By analyzing the core microbiome in all four treatments of soybean plants, we identified a total of 220 taxa, including 14 unique bacterial phyla. All bacterial phyla *Proteobacteria* were most abundant with 114 unique ASVs, followed by *Bacteroidota* with 24 unique ASVs, *Actinobacteriota Bdellovibrionota Myxococcota* with 17, 15, and 14 ASVs. Similarly, in core microbiome genera level analysis, we found 179 ASVs having 88 unique. Among all the genera, the highly abundant genera were *Edaphobacter* (12 unique ASVs)*, uncultured* (12 unique ASVs)*, Haliangium* (9 unique ASVs*)*, and *Streptomyces* (6 unique ASVs). To visualize the unique and shared core microbiomes, the Venn diagram analysis of the microbial ASVs was performed (**Figure 5**). The results revealed 57 ASVs as shared core-microbiome (bacterial), whereas some unique ASVs were also identified for each treatment. Among all the treatments, the Si+Cold treatment showed the highest unique ASVs (17), followed by the cold treatment (9 ASVs). Similarly, control Si-treated plants showed 7 and 4 unique ASVs, respectively (**Figure 5**). Furthermore, 14 ASVs were shared by Si, cold, and Si+Cold treatments. Control treatment shared the least ASVs with the other three treatments. Surprisingly, no fungal ASVs were detected in all treatments using the same set of analyses (**Supplementary Tables S12, S13**) (**Figure 5**). We also performed the Canonical Correspondence Analysis of the correlation of treatments vs. the extra-cellular enzyme interactions, which showed that Si with or without cold stress conditions forms a distinctive pattern compared to control and cold treatments (**Figure 5**).

### Interaction of microbial enzymes with silicon and cold stress in the rhizosphere

**Figure 6:**
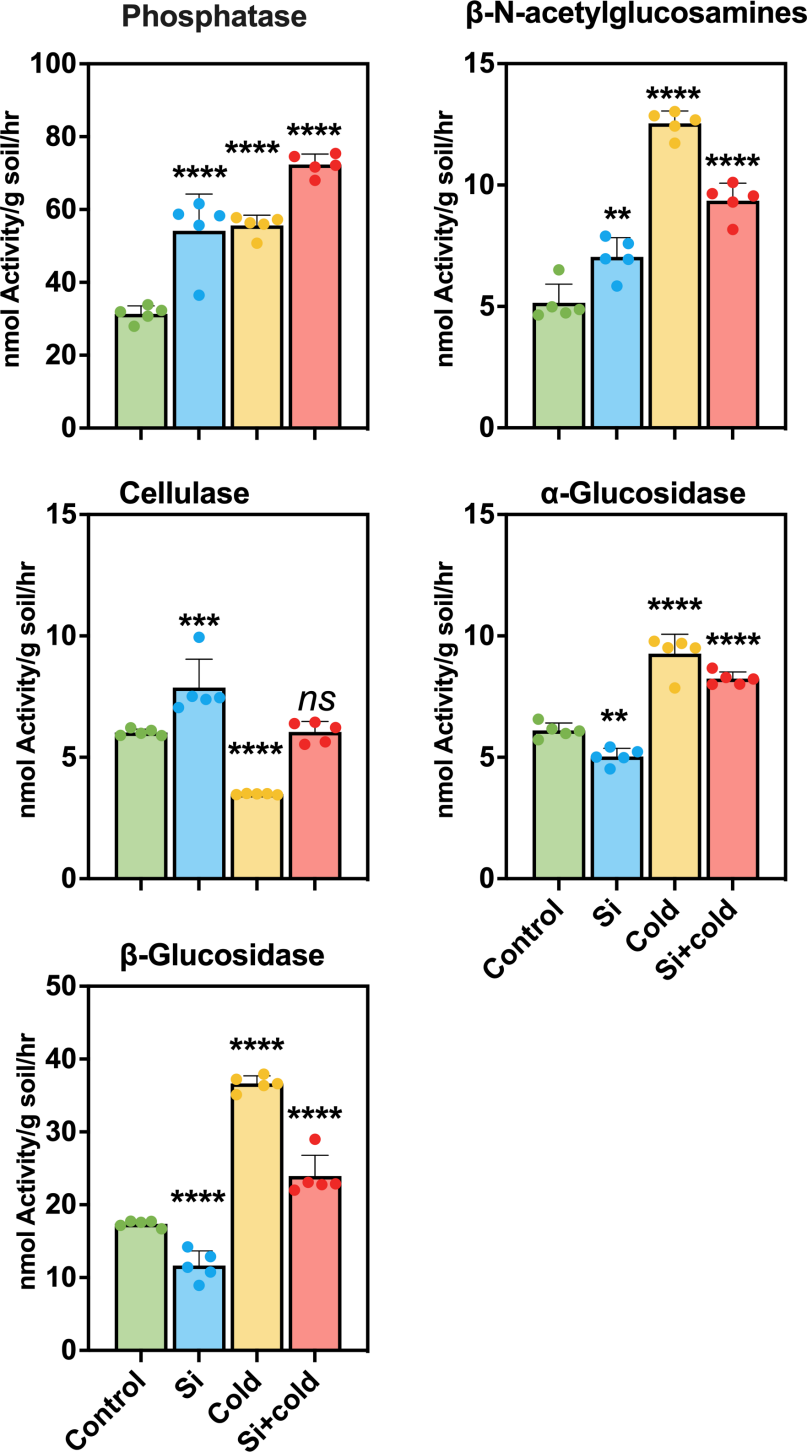
Extracellular enzymatic activities in rhizospheric soil of soybean plants cold stress and control conditions with and without exogenous supplementation of Si. The values represent the mean values of five replicates and show standard deviation. The bars showing **, ***, and **** are significantly different in their content compared to the control as analyzed by one-way ANOVA analysis. ‘ns’ shows a nonsignificant difference compared to the control.

We elucidated the activity of extracellular microbial enzymes in the soil to show how Si and cold stress influence microbial function in the rhizospheric soil. For this purpose, we assessed the enzymatic activities of Phosphatase (Phos), β-N-acetylglucosamines (NAG), Cellulase (CL), α-Glucosidase (AG), and β-Glucosidase (BG) in the soil samples of all treatments. The results showed that the Phos activity was highly significant (*p<*0.0001) in the soil of all treatments; the highest Phos activity was observed in the soil of Si+Cold treated plants (10.32 fold increase). Similarly, the Phos activity was increased by 73% in the soil of Si-treated plants. At the same time, the Phos activity in cold-stress plants was highly significant (*p<*0.0001) as compared to the control plants (78% increase). Similarly, the NAG activity observed in cold-treated plant soil was highly significant (*p<*0.0001) and highest compared to the other treatments and control (140% increase). The NAG activity in silicon-treated and Si+Cold-treated rhizospheric soil was also highly significant, and an increase of 40% and 80%, respectively, was observed compared to control plants’ rhizospheres. Interestingly, the activity of CL reduced significantly (*p<*0.0001) in cold-treated rhizospheres (34% decrease) compared to the control. At the same time, Si treatment increased BG enzymes’ activity in control and cold stress. In Si-treated plant rhizosphere, the NAG activity was increased by 30% compared to the control and 73% in Si+Cold treated compared to Cd-treated plant rhizospheres (**Figure 6**). Furthermore, the AG and BG activities showed an almost similar pattern; Si treatment reduced the enzyme activities significantly compared to the control, while the cold-treated plants’ rhizosphere exhibited a highly significant (*p<*0.0001) increase in both enzymatic activities. However, a highly significant increase was observed in AG (36%) and BG (35%) activities in Si+Cold treated plants’ rhizospheres compared to the control.

## Discussion

Soybean (*Glycine max)* is the world’s leading bioenergy and food crop and is highly sensitive to climate change, specifically cold stress at early growth stages (Kim et al., 2015). Plants sense and transmit cold signals to their cellular machinery using various mechanisms such as ROS (reactive oxygen species) production, calcium signaling, hormone production, and stress-related gene expression (Robison et al., 2019). The current study showed that cold stress reduced soybean plants’ overall growth. Several studies have shown that cold stress can reduce plant biomass by up to 18%. In the current study, we found that Si application maintained the plant growth and biomass during cold stress (Moradtalab et al., 2018a), possibly because Si can form phytoliths and subcutaneous layers, which can help the plants to reduce dehydration and maintain water turgor potential. Similar ameliorative effects were observed when Si was applied to *Phoenix dactylifera L.* and maize during cold stress by promoting plant biomass and growth (root and shoot lengths) (Moradtalab et al., 2018b; Bilal et al., 2023).

Moreover, Si application improved growth attributes in soybean plants under various biotic stresses like heat stress (Sharifi et al., 2022), water limitation (Sah et al., 2022), and low light (Sah et al., 2022). The temperature variation also impacts plant physio-biochemical parameters. The reactive oxygen species (ROS) formation has an immediate effect, further reducing cellular functionality and restricting growth and development (Sies and Jones, 2020). In response, the plant activates the defense system by increasing the antioxidant enzymes to eliminate ROS. For example, SOD, CAT, POD, and PPO promote alleviating the plant’s oxidative stress damage (Ren et al., 2023). It has been shown that Si plays an important role in mitigating cold stress-related oxidative damage (Zhu et al., 2004; Kim et al., 2017). We found that cold stress significantly increased the production of antioxidant enzymes than the control. Contrarily, the Si with cold stress application to soybean plants showed significantly lower enzyme activities – suggesting the mitigative function of Si against cold stress-induced ROS cellular influx in the phyllosphere and rhizosphere (Mir et al., 2022a). We observed significantly higher CAT activity in cold treatment than in control, whereas the Si application significantly reduced it during cold stress. A similar example of *Phyllostachys praecox* is highly susceptible to cold stress and exhibits elevated SOD, CAT, and POD activities with the Si treatment (Qian et al., 2019). Similarly, the SOD, POD, and PPO activities were higher in cold stress than in control, where the activities of the enzymes were reduced significantly in Si with a cold treatment (Prasad, 1997; Møller et al., 2007; Si et al., 2018; Tripathi et al., 2022) – revealing reducing low-temperature stress effects (Sharifi et al., 2022).

The Si influenced several key stress signaling-related genes in soybean plants under cold conditions. We assessed the gene expression of ABA-biosynthesis-related genes (*GmNCED3*, *ABAR1*, and *ABAR2*), dehydration (*GmDREB2A*) and abiotic stress (*GmWRKY27*, and *GmWRKY40*). ABA responds to environmental cues that modulate plant water or turgor potentials. In synergy, cold stress causes cellular desiccation and osmotic imbalance – enhancing expression patterns of ABA-related genes. *GmNCED3 –* major ABA biosynthesis-related gene, we found a 4.8-fold gene expression pattern in cold stress, significantly reduced (2.5 fold) in Si with cold stress (Qian et al., 2019). The ABA-receptor proteins *ABAR1* and *ABAR2* genes are involved in ABA biosynthesis and the circadian clock (Rane et al., 2021). Their expressions were significantly higher during cold stress, while, interestingly, Si reduced the *ABAR1* in the Si+Cold treatment. The relative expression of *GmWRKY27* improves abiotic stress tolerance in plants (Wang et al., 2015). Current results indicate the *GmWRKY27* expression increased significantly in cold and Si+Cold treatments; however, the expression level was comparatively lower in Si+Cold treatment. This indicates that Si application can reduce the oxidative stress caused by the cold conditions (Imran et al., 2021). Moreover, the *GmDREB2A* gene involves many pathways related to dehydration and water potential during stress (Ma et al., 2009). Our results showed that *GmDREB2A* was increased significantly in cold stress, and Si reduced its expression levels more than in cold stress. Dai et al. (2007) and Ma et al. (2009) showed that *GmDREB2A* was upregulated in cold stress in rice and *Arabidopsis*. Furthermore, Si has also been reported to upregulate the DREB2 gene along with other transcription factors to maintain the normal osmotic potentials in plant cells (Arif et al., 2021). The current result showed that upregulation of *GmDREB2A* in Si+Cold treatment than control, indicating a stress-protective role of Si. Overall, the *GmNCED3* gene expression levels were significantly higher than the *GmDREB2A* – suggesting a more potent role in cold stress resistance.

In synergy with soybean plant molecular responses, the microbial communities associated with plants can help plants cope with stressful conditions. In this case, the microbiome diversity can be reduced or increased to support plant growth. Our results showed that the beta-diversity demonstrated significant variations in the rhizosphere and phyllosphere of control vs. Si and Si vs. cold stress. The bacterial microbiome’s Shannon diversity was higher than fungal microbiomes across the treatments. However, the distribution of fungal species across the treatments was insignificant, whereas the distribution was significant compared to the bacterial microbiome across the plant compartments. The distribution of fungal species richness across plant compartments, especially soil, was also reported previously (Shen et al., 2021). To find out the cold-induced variations in cold vs. control and Si vs. control, the cold conditions reduced the bacterial diversity in the soil and fungal diversity in the leaf and root than in control. The current results of low diversity variations among treatments suggest that the cold conditions reduced the overall diversity. In previous reports, the plant microbiomes are directly impacted by external climatic conditions and indirectly by plant responses such as plant morphology, antioxidant defense, and genetics (Trivedi et al., 2022; Agyekum et al., 2023; Yang et al., 2023).

Similarly, reduced diversity in different plant parts due to cold stress was noted, suggesting the recruitment of specialized microbiota to cope with the stress condition (Marian et al., 2022). Therefore, the abundance of various microbial phyla was changed in different treatments. Similarly, Si did not impact the alpha and beta diversities dramatically but helped plants to increase diversity and recruit specialized bacterial and fungal communities in different compartments with or without stress. By elucidating bacterial and fungal communities’ abundances in the cold vs. control with and without Si treatments in soil, root, and shoot parts, we found that *Proteobacteria Actinobacteria*, *Bacteroidota*, and *Firmicutes* were significantly abundant. The *Gemmatimonadota*, *Chloroflexi*, and *Planctomycetota* were more abundant in the soil under cold stress, Si, and Si+Cold treated plants. Interestingly, multiple studies conducted on plant-associated microbial communities in Alpine and Arctic regions also found similar bacterial phyla (Nissinen et al., 2012; Kumar et al., 2017; Given et al., 2020; Marian et al., 2022). The exogenous supplementation of Si promotes plant growth, promoting bacterial and fungi (Wainwright et al., 1997; Ferrusquía-Jiménez et al., 2022), resulting in improved growth attributes and an abundance of specialized bacterial and fungal phyla in Si and Si+Cold treatments. Among the fungal communities, *Ascomycota* and *Basidiomycota* were more abundant in the different plant compartments, especially in stressed soil, than in root and leaf parts. However, Ascomycota was abundant in all treatments’ roots and soil. A recent report of *Ericaceae* species growing in Arctic regions showed that *Ascomycota* and *Basidomycota* were highly abundant (Wainwright et al., 1997; Zhang and Yao, 2015; Ferrusquía-Jiménez et al., 2022). A study conducted on the cultivable microbiome of the medicinal plant *Arnebia euchroma* growing in extreme cold and arid conditions of the Himalayas detected the highest abundances of *Proteobacteria* and *Ascomycota* (Jain et al., 2021).

Soybean plants’ leaf and root compartments showed increased genus-level diversity compared to soil. It is because the leaf and root parts are directly affected by the environmental stimulus caused by cold stress, resulting in morphological, biochemical, and molecular changes (Zhu et al., 2022). Among the bacterial genera, the cold-treated plant’s leaf and root compartments recruited the *Pseudomonas*, *Massilia, and Bacillus* in the leaf and root compartments as compared to the control, and these genera are well known for their plant growth-promoting activities (Preston, 2004; Radhakrishnan et al., 2017; Holochová et al., 2020) Si-treated root and soil in cold (Si+Cold) and normal (Si) conditions showed an increased abundance of *Noviherbaspirillum* and *Bacillus* species respectively. The *Noviherbaspirillum* has previously been reported in lettuce cold-specific root microbiome (Persyn et al., 2022) and other extreme environments like drought (Khan et al., 2022a), and it has plant stress tolerance promoting properties (Zhang et al., 2021). Whereas the *bacillus* is known as Si solubilizing bacteria (SSB) and has been documented multiple times for its role in solubilizing the Si from complex forms and making it available for plant uptake (Vasanthi et al., 2018; Etesami and Jeong, 2022). *Bacillus* is found abundant in Si-treated cold stress root and soil. The *Sphingomonas* species were more abundant in leaf and root compartments in Si and Si+Cold treatments and have also been reported previously for enhancing cold stress tolerance in tomato plants (Subramanian et al., 2016; Moroenyane et al., 2021).

In fungal communities, the *Alternaria* genus was more abundant in cold-treated leaf parts than in control plants. However, the *Alternaria* species have pathogenic properties (Attia et al., 2020; Boyno et al., 2022). Recently, their plant growth-promoting activities are also reported (Zhou et al., 2018; Mauricio-Castillo et al., 2020). Moreover*, Fusarium* and *Gibberella* genera were more abundant in the roots of cold-treated plants than control. *Pseudomonas* sp. *was* reported to regulate the cold-induced freezing tolerance gene *SEX1* in the roots of *N. tobaccum* and *A. thaliana* (Lata et al., 2018). The *Fusarium* and *Gibberalla* are known for their gibberellins-producing properties and for promoting plants’ abilities to survive abiotic stress conditions (Bilal et al., 2018). *Burkholderia* genus, previously known to assist plants in solubilization and uptake of Si, was more abundant in cold and Si+Cold treatments (Kang et al., 2017). The abundance of *Burkholderia* in cold stress with and without silicon explains its role in promoting cold tolerance in soybean plants. The interaction of a single bacterial genus (*Variovorax*) reversed root inhibition by manipulating plant hormones to balance the adverse effects of the stress (Finkel et al., 2020).

The microbiome composition is directly proportional to the extracellular enzyme activities in the soil or rhizosphere parts. Extracellular soil enzymes break down different macromolecules like sugar-based polymers, carbohydrates, lignin, proteins, and organic acids to macromolecules transported across the plant cells. Most of these enzymes are produced by microbiome species. The enzymes not just promote plant growth and development but also has a direct impact on the plant-microbe symbiosis (Eid et al., 2019). For example, the insoluble cation-bound phosphate complexes are solubilized by phosphatases, making them accessible for plant uptake (Ndabankulu et al., 2022). We assessed the activities of extracellular enzymes, i.e., Phos, NAG, CL, AG, and BG. In this study, the exogenous application of silicon in cold conditions (Si+Cold) increased the phosphatase activity significantly. Similarly, the enzymes NAG, AG, and BG were decreased in cold-treated soil than in Si and Si+Cold treatments. Interestingly, Si enhances enzymatic activities in different abiotic stress conditions like salt and heavy metals (Bhalla and Garg, 2021; Mir et al., 2022b). Our results of a high abundance of *Ascomycota* and *Basidiomycete* can be correlated to increased plant growth and production of extracellular enzymes. Previously the *Proteobacteria, Bacteroidetes, Actinobacteria*, *Firmicutes*, *Planctomycetes*, and *Chloroflexi* were reported for their positive impact on extracellular soil enzymes (Li et al., 2019; Bhalla and Garg, 2021).

## Conclusion

In the face of increasing global climate change, there is a growing need to incorporate plant-microbe symbioses to enhance plant growth and stress tolerance in agriculture ecosystems. The present study showed that cold stress exposures adversely impacted and regulated the plant growth, physiology, and expression of critical genes involved in the plant defense system. Si application has improved plant growth and stress responses by modulating the antioxidant defense system and regulating stress-responsive genes (osmoregulatory and ABA biosynthesis-related genes). The findings also showed that cold stress impacts soybean plants’ rhizospheric and phyllospheric diversity. Overall, cold stress impacted the bacterial diversity across the treatments (Si and cold) and plant parts (soil, root, and shoot); however, the fungal communities were less diverse across the treatments where the fungal compositions were prominent across the plant parts. The microbial diversity was also correlative to the extracellular soil enzyme activities.

In conclusion, the exogenous Si supplementation improved the cold stress tolerance in soybean plants and assisted in recruiting specialized microbiome players. This study will shed light on understanding the cold-tolerant microbial players. The finding will contribute towards understanding the dynamic and complex of plant-microbe-stress interaction. Since Si is a beneficial element for plant growth, this can be used as a climate-smart intervention to protect plant growth during climate change episodes. However, broader field trials in agricultural and mixed-community settings will further understand the beneficial role of Si in agroecosystems’ soil, plant, and microbial health. Studies at a broader field scale across different spatial-temporal conditions of stress (type, intensity, and duration) with or without the presence of Si can better understand plant stress tolerance and microbiome diversity function. Future studies can focus more on large-scale metagenome shotgun sequencing, and analysis can help elucidate the underlying mechanisms and processes that microbes use during stressful conditions.

## Data availability statement

The datasets presented in this study can be found in NCBI, the accession number(s) are Bioproject PRJNA981149 and are available under SRR24887345-SRR24887340 (ITS), SRR24880681-SRR24880652 (16S).

## Author contributions

WA: Data curation, Investigation, Validation, Visualization, Writing – original draft. LC: Data curation, Software, Visualization, Writing – review & editing. AW: Funding acquisition, Resources, Validation, Writing – review & editing. KC: Investigation, Resources, Writing – review & editing. AK: Conceptualization, Funding acquisition, Project administration, Supervision, Writing – review & editing, Investigation.

## Supplementary material

Supplementary Figure 1 Chlorophyll Contents and Growth Attributes of soybean plants. The bars showing *, **, ***, and **** are significantly different in their content compared to the control as analyzed by one-way ANOVA analysis. ‘ns’ shows a nonsignificant difference compared to the control.

Supplementary Figure 2 Principal coordinates analysis (PCoA) based on the soybean plant’s Bray-Curtis distances of the bacterial and fungal biome. **(A–C)** PCoA of Bray-Curtis of bacterial biomes. **(D–F)** PCoA of Bray-Curtis of fungal biomes.

Supplementary Figure 3 Comparison of the Genus-level distribution of soybean plants’ bacterial and fungal biome. **(A)** Genus-level distribution of bacterial biome in the cold vs. control. **(B)** Genus-level distribution of bacterial biome in Si vs. control. **(C)** Genus-level distribution of fungal biome in the cold vs. control. **(D)** Genus-level distribution of fungal biome in Si vs. control. CT.L, CT.R, and CT.S are control leaf, root and soil, respectively. CD.L, CD.R, and CD.S are cold-treated leaf, root and Soil, respectively. SI.L, SI.R. SI.S are Si-treated leaf, root and soil respectively. SI+CD.L, SI+CD.R and SI+CD.S are Si+Cold leaf, root and soil, respectively.

Supplementary Figure 4 Comparison of the Phylum-level distribution of the bacterial and fungal biome across the different plant compartments in all treatments. **(A)** Phylum-level bacterial biome distribution in Leaf **(B)** Phylum-level bacterial biome distribution in Root. **(C)** Phylum-level bacterial biome distribution in Soil. **(D)** Phylum-level fungal biome distribution in Leaf. **(E)** Phylum-level fungal biome distribution in Root. **(F)** Phylum-level fungal biome distribution in Soil. CD.L, CD.R, and CD.S are cold-treated leaf, root and Soil, respectively. SI.L, SI.R. SI.S are Si-treated leaf, root and soil respectively. SI+CD.L, SI+CD.R and SI+CD.S are Si+Cold leaf, root and soil, respectively.

Supplementary Figure 5 Comparison of the Genus-level distribution of the bacterial and fungal biome across the different plant compartments in all treatments. **(A)** Genus-level bacterial biome distribution in Leaf **(B)** Genus-level bacterial biome distribution in Root. **(C)** Genus-level bacterial biome distribution in Soil. **(D)** Genus-level fungal biome distribution in Leaf. **(E)** Genus-level fungal biome distribution in Root. **(F)** Genus-level fungal biome distribution in Soil. CD.L, CD.R, and CD.S are cold-treated leaf, root and Soil respectively. SI.L, SI.R. SI.S are Si-treated leaf, root and soil respectively. SI+CD.L, SI+CD.R and SI+CD.S are Si+Cold leaf, root and soil, respectively.
