# Supplementary material for: The silicon regulates microbiome diversity and plant defenses during cold stress in Glycine max L
Source: Front Plant Sci. 2024 Jan 10;14:1280251. doi: 10.3389/fpls.2023.1280251 (PMC10805835; doi:10.3389/fpls.2023.1280251)
Supplement: Supplementary file 1 [file DataSheet_1.zip › Supplementary Figures.pdf]

The silicon regulates microbiome diversity and plant defenses during cold stress in *Glycine max* L.

Waqar Ahmad[1,3], Lauryn Coffman[1], Aruna Weerasooriya[2], Kerri Crawford[3], Abdul Latif Khan[1,3]*

[1]Department of Engineering Technology, Cullen College of Engineering, University of Houston, Sugar Land, TX, USA

[2]Cooperative Agricultural Research Center, College of Agriculture & Human Sciences, Prairie View A&M University, Prairie View, TX, USA

[3]Department of Biology and Biochemistry, College of Natural Science & Mathematics, University of Houston, Houston, TX, USA

***Corresponding author:**

Abdul Latif Khan; alkhan@uh.edu

Supplementary Figures

Figure S1

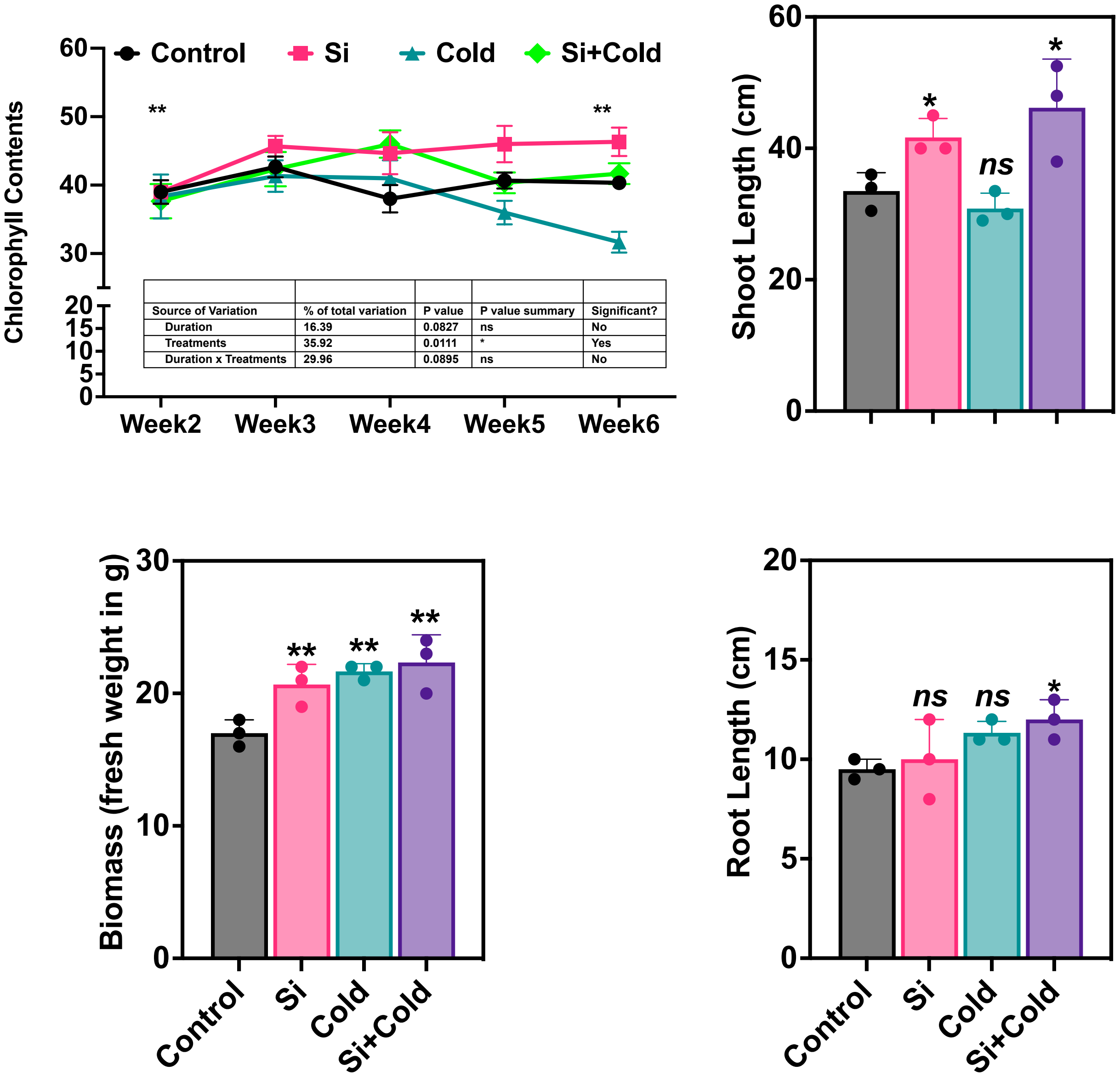

Source of Variation	% of total variation	P value	P value summary	Significant?
Duration	16.39	0.0827	ns	No
Treatments	35.92	0.0111	*	Yes
Duration x Treatments	29.96	0.0895	ns	No

Figure S2

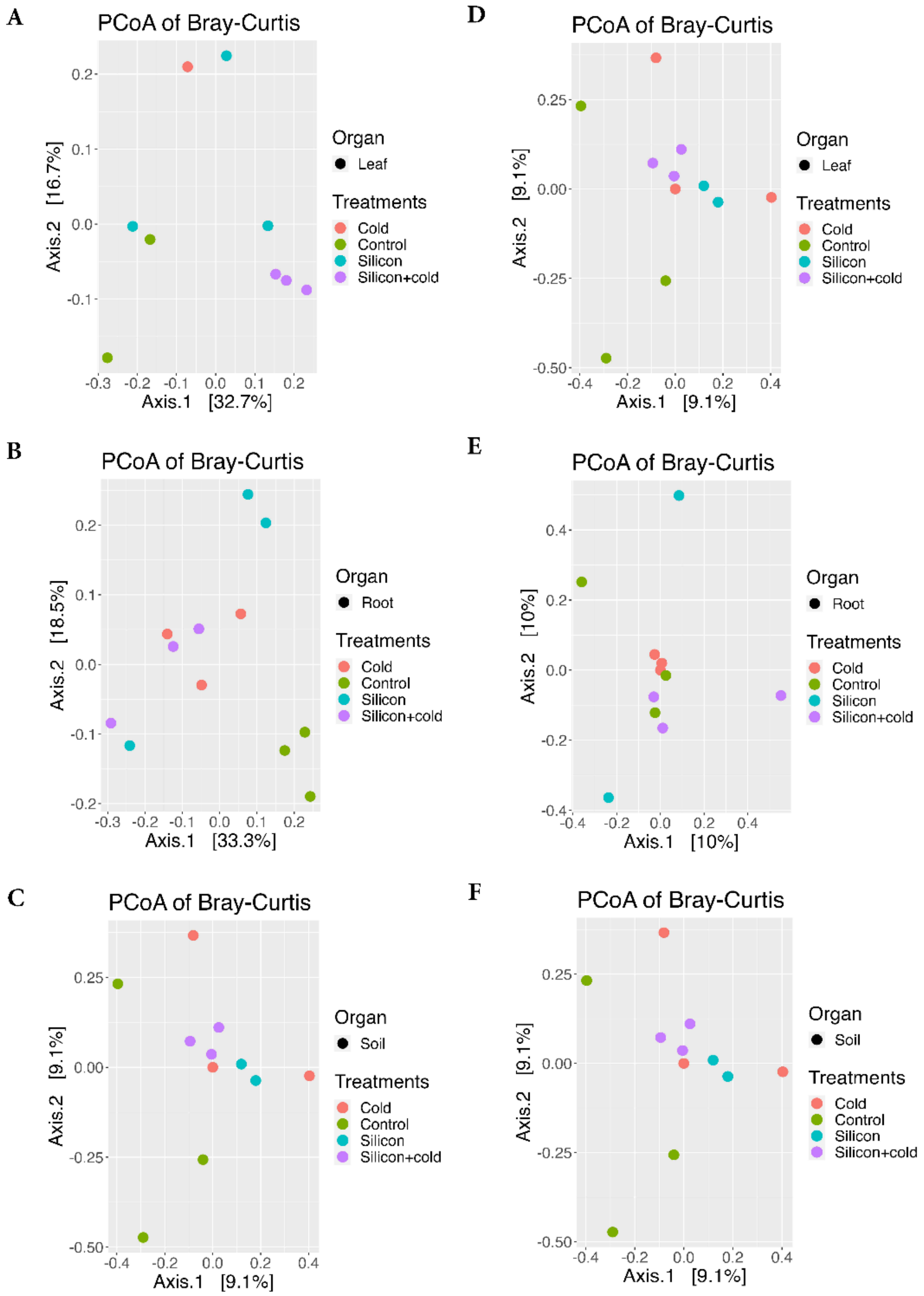
A
PCoA of Bray-Curtis
Axis.1 [32.7%]
Axis.2 [16.7%]
Organ
Leaf
Treatments
Cold
Control
Silicon
Silicon+cold
B
PCoA of Bray-Curtis
Axis.1 [33.3%]
Axis.2 [18.5%]
Organ
Root
Treatments
Cold
Control
Silicon
Silicon+cold
C
PCoA of Bray-Curtis
Axis.1 [9.1%]
Axis.2 [9.1%]
Organ
Soil
Treatments
Cold
Control
Silicon
Silicon+cold
D
PCoA of Bray-Curtis
Axis.1 [9.1%]
Axis.2 [9.1%]
Organ
Leaf
Treatments
Cold
Control
Silicon
Silicon+cold
E
PCoA of Bray-Curtis
Axis.1 [10%]
Axis.2 [10%]
Organ
Root
Treatments
Cold
Control
Silicon
Silicon+cold
F
PCoA of Bray-Curtis
Axis.1 [9.1%]
Axis.2 [9.1%]
Organ
Soil
Treatments
Cold
Control
Silicon
Silicon+cold

Figure S3

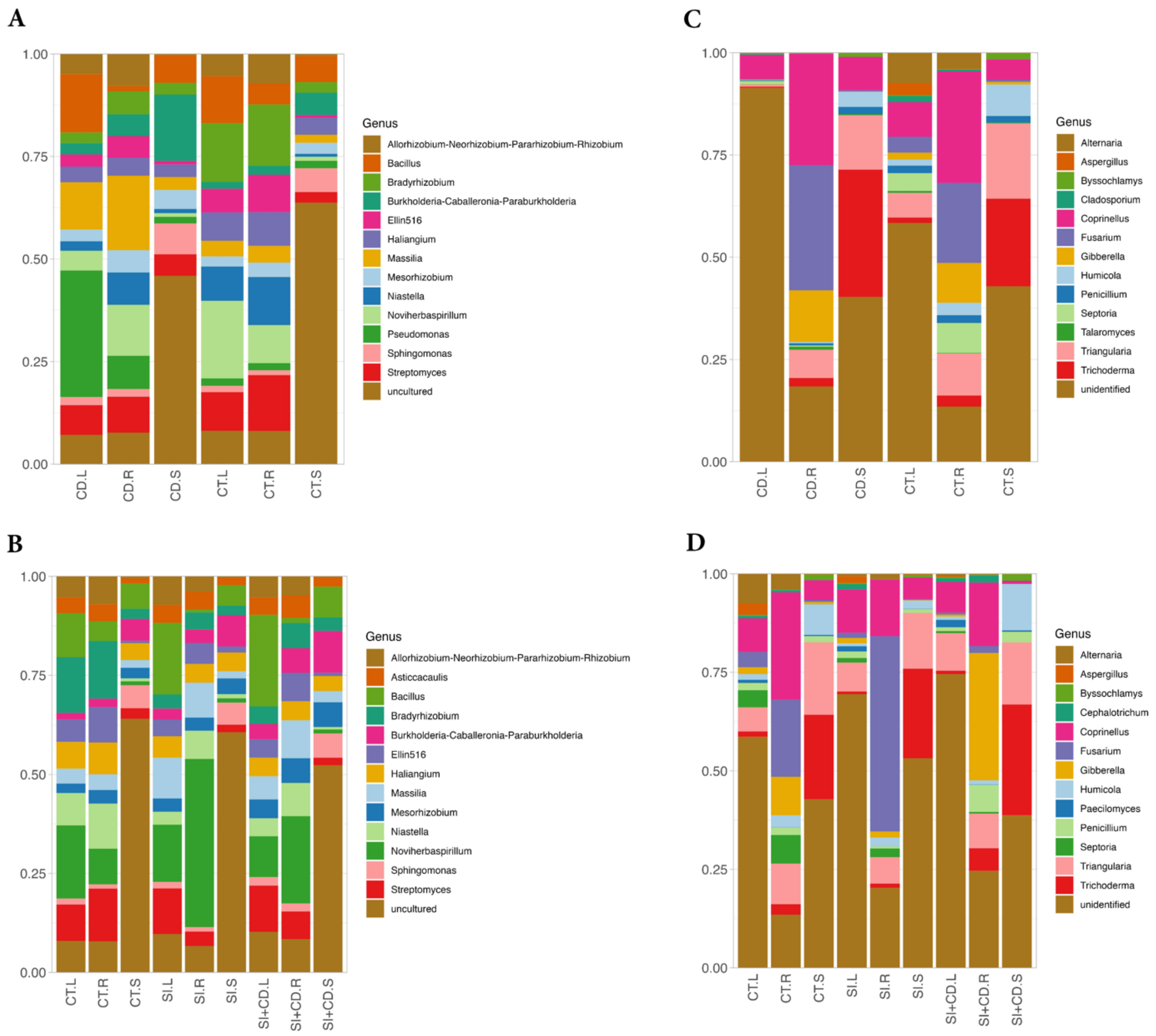
A
1.00
0.75
0.50
0.25
0.00
CD.L
CD.R
CD.S
CT.L
CT.R
CT.S
Genus
Allorhizobium-Neorhizobium-Pararhizobium-Rhizobium
Bacillus
Bradyrhizobium
Burkholderia-Caballeronia-Paraburkholderia
Ellin516
Haliangium
Massilia
Mesorhizobium
Niastella
Noviherbaspirillum
Pseudomonas
Sphingomonas
Streptomyces
uncultured
C
1.00
0.75
0.50
0.25
0.00
CD.L
CD.R
CD.S
CT.L
CT.R
CT.S
Genus
Alternaria
Aspergillus
Byssochlamys
Cladosporium
Coprinellus
Fusarium
Gibberella
Humicola
Penicillium
Septoria
Talaromyces
Triangularia
Trichoderma
unidentified
B
1.00
0.75
0.50
0.25
0.00
CT.L
CT.R
CT.S
SI.L
SI.R
SI.S
SI+CD.L
SI+CD.R
SI+CD.S
Genus
Allorhizobium-Neorhizobium-Pararhizobium-Rhizobium
Asticcacaulis
Bacillus
Bradyrhizobium
Burkholderia-Caballeronia-Paraburkholderia
Ellin516
Haliangium
Massilia
Mesorhizobium
Niastella
Noviherbaspirillum
Sphingomonas
Streptomyces
uncultured
D
1.00
0.75
0.50
0.25
0.00
CT.L
CT.R
CT.S
SI.L
SI.R
SI.S
SI+CD.L
SI+CD.R
SI+CD.S
Genus
Alternaria
Aspergillus
Byssochlamys
Cephalotrichum
Coprinellus
Fusarium
Gibberella
Humicola
Paecilomyces
Penicillium
Septoria
Triangularia
Trichoderma
unidentified

Figure S4

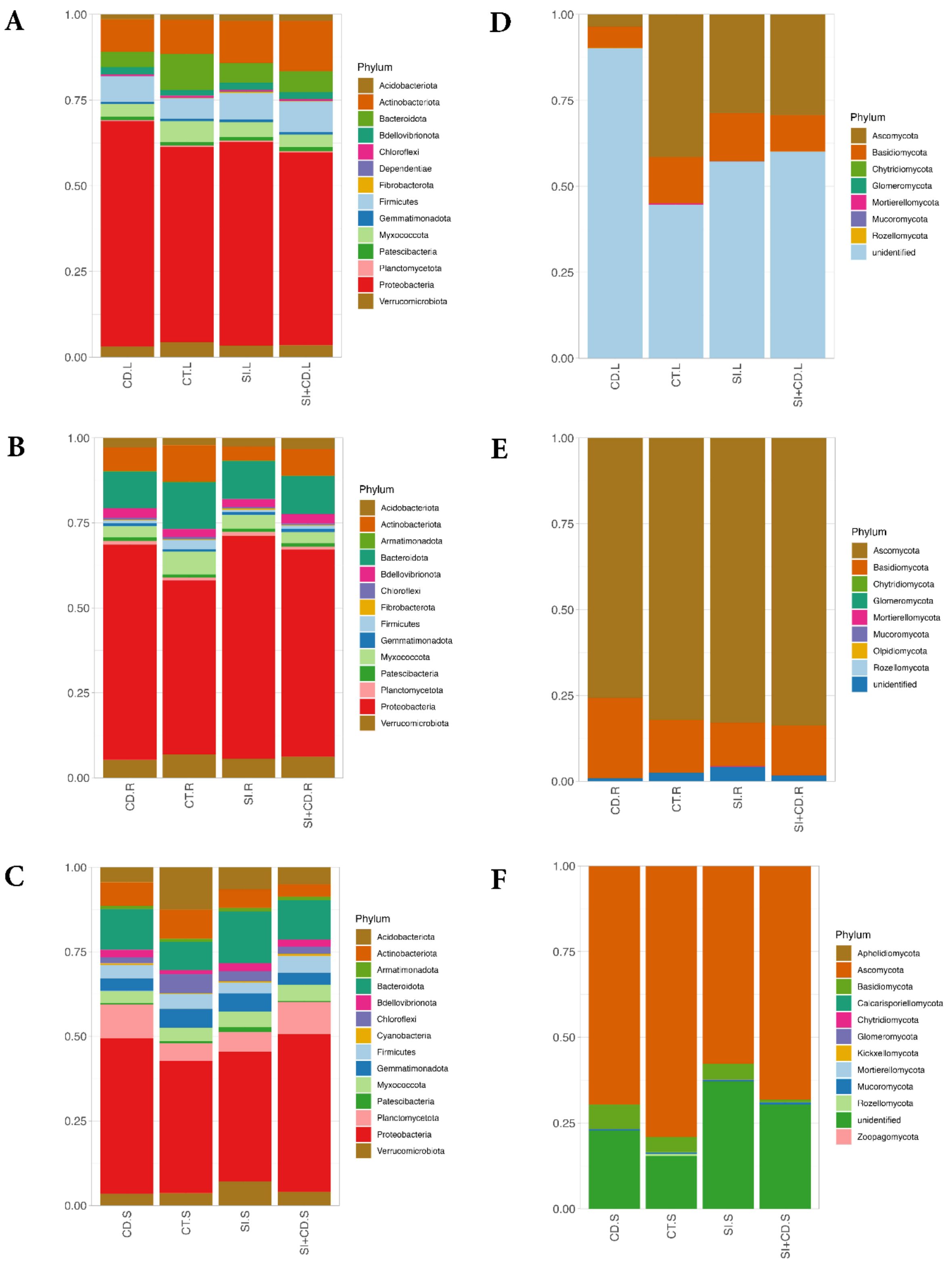
A
1.00
0.75
0.50
0.25
0.00
CD.L
CT.L
SI.L
SI+CD.L
Phylum
Acidobacteriota
Actinobacteriota
Bacteroidota
Bdellovibrionota
Chloroflexi
Dependentiae
Fibrobacterota
Firmicutes
Gemmatimonadota
Myxococcota
Patescibacteria
Planctomycetota
Proteobacteria
Verrucomicrobiota
B
CD.R
CT.R
SI.R
SI+CD.R
Phylum
Acidobacteriota
Actinobacteriota
Armatimonadota
Bacteroidota
Bdellovibrionota
Chloroflexi
Fibrobacterota
Firmicutes
Gemmatimonadota
Myxococcota
Patescibacteria
Planctomycetota
Proteobacteria
Verrucomicrobiota
C
CD.S
CT.S
SI.S
SI+CD.S
Phylum
Acidobacteriota
Actinobacteriota
Armatimonadota
Bacteroidota
Bdellovibrionota
Chloroflexi
Cyanobacteria
Firmicutes
Gemmatimonadota
Myxococcota
Patescibacteria
Planctomycetota
Proteobacteria
Verrucomicrobiota
D
CD.L
CT.L
SI.L
SI+CD.L
Phylum
Ascomycota
Basidiomycota
Chytridiomycota
Glomeromycota
Mortierellomycota
Mucoromycota
Rozellomycota
unidentified
E
CD.R
CT.R
SI.R
SI+CD.R
Phylum
Ascomycota
Basidiomycota
Chytridiomycota
Glomeromycota
Mortierellomycota
Mucoromycota
Olpidiomycota
Rozellomycota
unidentified
F
CD.S
CT.S
SI.S
SI+CD.S
Phylum
Aphelidiomycota
Ascomycota
Basidiomycota
Calcarisporiellomycota
Chytridiomycota
Glomeromycota
Kickxellomycota
Mortierellomycota
Mucoromycota
Rozellomycota
unidentified
Zoopagomycota

Figure S5

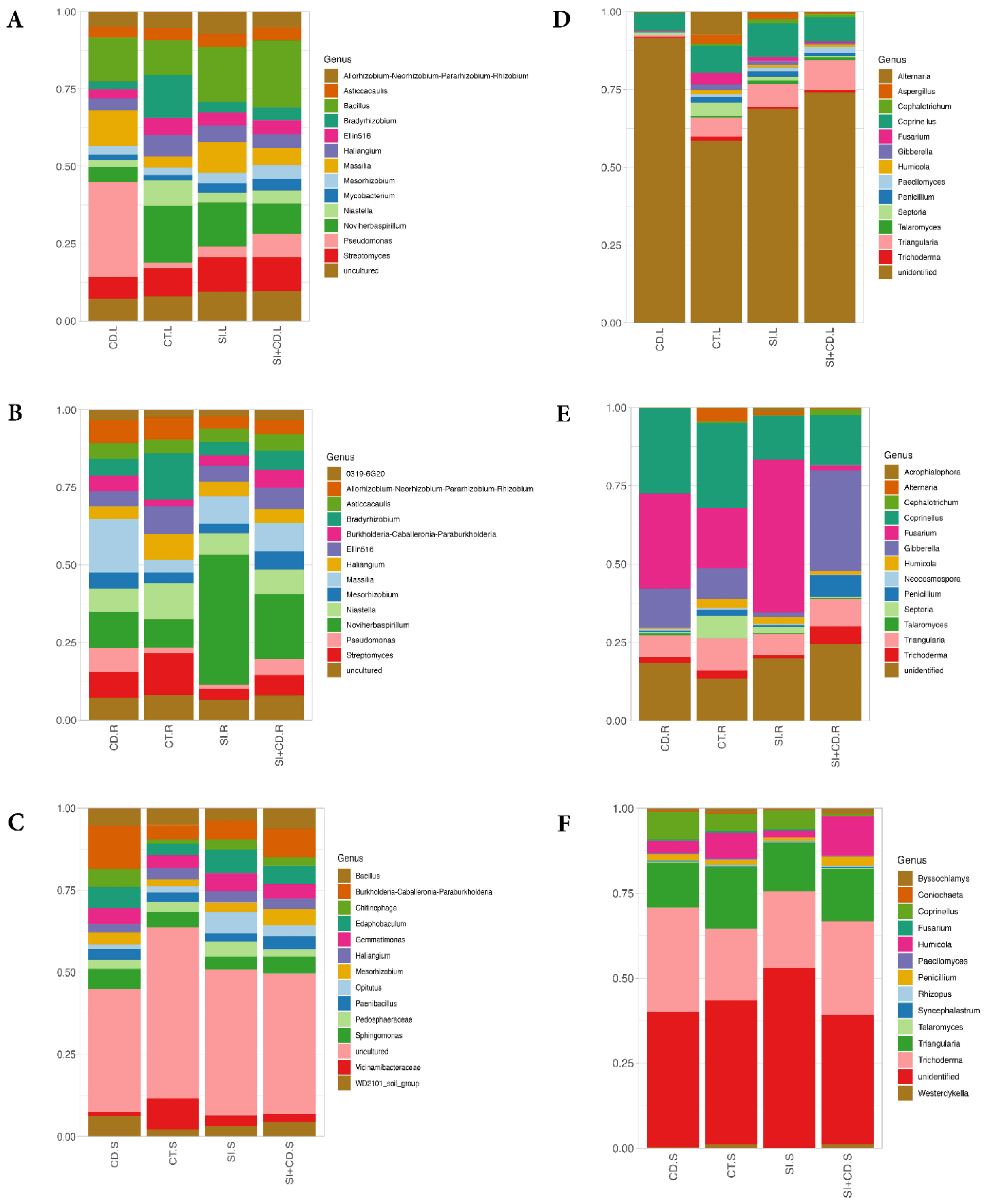
A
1.00
0.75
0.50
0.25
0.00
CD.L
CT.L
SI.L
SI+CD.L
Genus
Allorhizobium-Neorhizobium-Pararhizobium-Rhizobium
Asticcacaulis
Bacillus
Bradyrhizobium
Ellin516
Haliangium
Massilia
Mesorhizobium
Mycobacterium
Niastella
Noviherbaspirillum
Pseudomonas
Streptomyces
uncultured
D
1.00
0.75
0.50
0.25
0.00
CD.L
CT.L
SI.L
SI+CD.L
Genus
Alternaria
Aspergillus
Cephalotrichum
Coprinellus
Fusarium
Gibberella
Humicola
Paecilomyces
Penicillium
Septoria
Talaromyces
Triangularia
Trichoderma
unidentified
B
1.00
0.75
0.50
0.25
0.00
CD.R
CT.R
SI.R
SI+CD.R
Genus
0319-6G20
Allorhizobium-Neorhizobium-Pararhizobium-Rhizobium
Asticcacaulis
Bradyrhizobium
Burkholderia-Caballeronia-Paraburkholderia
Ellin516
Haliangium
Massilia
Mesorhizobium
Niastella
Noviherbaspirillum
Pseudomonas
Streptomyces
uncultured
E
1.00
0.75
0.50
0.25
0.00
CD.R
CT.R
SI.R
SI+CD.R
Genus
Acrophialophora
Alternaria
Cephalotrichum
Coprinellus
Fusarium
Gibberella
Humicola
Neocosmospora
Penicillium
Septoria
Talaromyces
Triangularia
Trichoderma
unidentified
C
1.00
0.75
0.50
0.25
0.00
CD.S
CT.S
SI.S
SI+CD.S
Genus
Bacillus
Burkholderia-Caballeronia-Paraburkholderia
Chitinophaga
Edaphobaculum
Gemmatimonas
Haliangium
Mesorhizobium
Opitutus
Paenibacillus
Pedosphaeraceae
Sphingomonas
uncultured
Vicinamibacteraceae
WD2101_soil_group
F
1.00
0.75
0.50
0.25
0.00
CD.S
CT.S
SI.S
SI+CD.S
Genus
Byssochlamys
Coniochaeta
Coprinellus
Fusarium
Humicola
Paecilomyces
Penicillium
Rhizopus
Syncephalastrum
Talaromyces
Triangularia
Trichoderma
unidentified
Westerdykella
