# Supplementary material for: The silicon regulates microbiome diversity and plant defenses during cold stress in Glycine max L
Source: Front Plant Sci. 2024 Jan 10;14:1280251. doi: 10.3389/fpls.2023.1280251 (PMC10805835; doi:10.3389/fpls.2023.1280251)
Supplement: Supplementary file 1 [file DataSheet_1.zip › Supplementary Tables.pdf]

The silicon regulates microbiome diversity and plant defenses during cold stress in *Glycine max* L.

Waqar Ahmad[1,3], Lauryn Coffman[1], Aruna Weerasooriya[2], Kerri Crawford[3], Abdul Latif Khan[1,3]*

[1]Department of Engineering Technology, Cullen College of Engineering, University of Houston, Sugar Land, TX, USA

[2]Cooperative Agricultural Research Center, College of Agriculture & Human Sciences, Prairie View A&M University, Prairie View, TX, USA

[3]Department of Biology and Biochemistry, College of Natural Science & Mathematics, University of Houston, Houston, TX, USA

***Corresponding author:**

Abdul Latif Khan; alkhan@uh.edu

Supplementary Data File

Table S1. Gene primers

Name	Sequence - 5’ to 3’	Length	GC	Tm (°C)
ACT11-F	ATCTTGACTGAGCGTGGTTATTCC	24	0.46	58.6
ACT11-R	GCTGGTCCTGGCTGTCTCC	19	0.68	64
GmNCED3-F	ATGGCGAGGAGTTTTCCGTTGAAGAAGA	28	0.46	62.5
GmNCED3-R	ATGGCGAGGAGTTTTCCGTTGAAGAAGA	28	0.46	62.5
GmWRKY27-F	CTTCTGGTGATTCAGTTTTGGGATT	25	0.4	57.3
GmWRKY27-R	ACGGATGAGGAACTGATTGTGT	22	0.45	56.2
GmDREB2A-F	CATGTGATCCACAGCCTCATCCTTA	24	0.46	58.6
GmDREB2A-R	GTAACAACAGGTTCCAACCGTTCA	25	0.4	57.3
ABAR1-F	GTATCTTCACCATTTACTCTTCCAAGC	27	0.41	59.3
ABAR1-R	GGTGAATAGGCCATTGCCAATGAC	24	0.5	60.4
ABAR2-F	CAAATCTGAACATTGCTTACAAAATGAATGTT	32	0.28	57.6
ABAR2-R	ATTACCATACTGTTTCCCATATACCAG	27	0.37	57.8
GmWRKY40-F	TGAGCAGGACCAGATACCTACAC	23	0.52	60.2
GmWRKY40-R	GAAGGTGCTATGGAACTAAAGGC	23	0.48	58.4

Table S2. Summary of sequencing data information of 16S leaf and root samples

Sample	RawPE	Combined	Qualified	Nochime	Base(nt)	Avglen(nt)	GC	Q20	Q30	Effective%
C.L1	156457	153426	150681	126426	47629468	376.74	54.64%	98.55%	94.88%	80.81%
C.L2	153579	150963	147976	133559	50289714	376.54	54.63%	98.63%	95.26%	86.96%
C.L3	153829	151166	148549	128585	48432961	376.66	54.62%	98.63%	95.25%	83.59%
SI.L1	154236	151740	148921	128065	48246904	376.74	54.62%	98.60%	95.16%	83.03%
SI.L2	152175	149379	146716	129514	48799440	376.79	54.72%	98.57%	95.10%	85.11%
SI.L3	154293	150959	147667	127598	48054636	376.61	54.69%	98.42%	94.56%	82.70%
CC.L1	154452	151936	148969	128117	48247406	376.59	54.62%	98.58%	95.08%	82.95%
CC.L2	158647	155130	152748	129596	48775489	376.37	54.80%	98.50%	94.83%	81.69%
CC.L3	156456	155783	154608	144879	54595908	376.84	54.72%	99.29%	97.19%	92.60%
SI.C.L1	155824	153389	150487	138470	52172623	376.78	54.63%	98.55%	95.02%	88.86%
SI.C.L2	152418	149362	146725	126927	47812065	376.69	54.69%	98.57%	95.11%	83.28%
SI.C.L3	155696	153401	150798	136098	51278539	376.78	54.66%	98.68%	95.40%	87.41%
C.R1	154082	151856	149116	79487	30071607	378.32	54.99%	98.50%	94.87%	51.59%
C.R2	152962	140622	137998	96830	36539656	377.36	54.67%	98.13%	93.72%	63.30%
C.R3	156725	129584	127101	97255	36671349	377.06	54.28%	98.36%	94.37%	62.05%
SI.R1	155482	130354	127770	111626	41998572	376.24	54.38%	98.47%	94.82%	71.79%
SI.R2	155644	127761	125544	99544	37363241	375.34	53.95%	98.42%	94.66%	63.96%
SI.R3	153801	134824	132242	101752	38258739	376	54.08%	98.36%	94.49%	66.16%
CC.R1	153410	128979	126627	101463	38202770	376.52	54.47%	98.43%	94.71%	66.14%
CC.R2	154377	126979	124268	99240	37332466	376.18	54.34%	98.39%	94.49%	64.28%
CC.R3	155120	137437	134510	98650	37123691	376.32	54.19%	98.20%	93.98%	63.60%
SI.C.R1	154769	129480	127170	112612	42390570	376.43	54.41%	98.52%	94.93%	72.76%
SI.C.R2	165603	136599	133978	105118	39561955	376.36	54.38%	98.47%	94.82%	63.48%
SI.C.R3	154696	117365	115317	92796	34921469	376.33	54.37%	98.48%	94.85%	59.99%

Table S3. Summary of sequencing data information of 16S soil samples

Sample	NovoID	Raw Reads	Clean Reads	Raw Base(G)	Clean Base(G)	Error Rate(%)	Q20(%)	Q30(%)	GC Content(%)
C.S1	FKDN220495909-1A	138526	138515	0.07	0.07	0.03	97.92	93.86	55.91
C.S2	FKDN220495910-1A	145163	145144	0.07	0.07	0.03	98.02	94.02	55.7
C.S3	FKDN220495911-1A	166809	166796	0.08	0.08	0.03	97.94	93.71	55.59
SI.S1	FKDN220495912-1A	189027	189010	0.09	0.09	0.03	98.01	93.91	55.24
SI.S2	FKDN220495913-1A	168206	168190	0.08	0.08	0.03	97.96	93.71	53.99
SI.S3	FKDN220495914-1A	165835	165820	0.08	0.08	0.03	97.92	93.72	54.15
CC.S1	FKDN220495915-1A	182592	182576	0.09	0.09	0.03	97.95	93.81	55.14
CC.S2	FKDN220495916-1A	171418	171376	0.09	0.09	0.03	97.95	93.84	54.78
CC.S3	FKDN220495917-1A	152266	152257	0.08	0.08	0.03	97.88	93.67	54.74
SI.C.S1	FKDN220495918-1A	117442	117433	0.06	0.06	0.03	97.99	93.98	54.96
SI.C.S2	FKDN220495919-1A	150072	150063	0.08	0.08	0.03	97.91	93.75	54.95
SI.C.S3	FKDN220495920-1A	145693	145683	0.07	0.07	0.03	97.84	93.61	54.49

Table S4. Summary of sequencing data information of ITS leaf, root and soil samples

Sample	NovoID	Raw Reads	Clean Reads	Raw Base(G)	Clean Base(G)	Error Rate(%)	Q20(%)	Q30(%)	GC Content(%)
C.L1	FKDN220495972-1A	149694	149532	0.07	0.07	0.03	95.61	89.41	47.93
C.L2	FKDN220495973-1A	181493	181416	0.09	0.09	0.03	95.74	89.73	52.18
C.L3	FKDN220495975-1A	187822	187773	0.09	0.09	0.03	96.39	90.72	53.8
SI.L1	FKDN220495976-1A	188682	188622	0.09	0.09	0.03	95.49	89.61	48.9
SI.L2	FKDN220495977-1A	181461	181427	0.09	0.09	0.03	96.16	89.93	46.02
SI.L3	FKDN220495979-1A	181059	181020	0.09	0.09	0.03	96.77	91.44	46.57
CC.L1	FKDN220495978-1A	152569	152517	0.08	0.08	0.03	96.6	90.61	57.42
CC.L2	FKDN220495980-1A	185614	185478	0.09	0.09	0.03	96.75	90.98	56.97
CC.L3	FKDN220495981-1A	186225	186194	0.09	0.09	0.03	95.91	89.91	52.86
SI.C.L1	FKDN220495982-1A	172046	172017	0.09	0.09	0.03	96.43	90.61	53.28
SI.C.L2	FKDN220495983-1A	159681	159308	0.08	0.08	0.03	95.85	89.82	51.88
SI.C.L3	FKDN220495984-1A	185878	185831	0.09	0.09	0.03	95.83	89.86	48.91
C.R1	FKDN220495974-1A	167711	167629	0.08	0.08	0.03	95.86	89.93	52.51
C.R2	FKDN220495985-1A	189628	189466	0.09	0.09	0.03	95.89	89.97	48.19

C.R3	FKDN220495986-1A	189442	189426	0.09	0.09	0.03	95.76	89.77	49.01
SI.R1	FKDN220495987-1A	156037	156028	0.08	0.08	0.03	96.42	90.84	47.81
SI.R2	FKDN220495988-1A	106149	106125	0.05	0.05	0.03	95.9	90.08	48.72
SI.R3	FKDN220495989-1A	177718	177710	0.09	0.09	0.03	95.88	90.42	47.59
CC.R1	FKDN220495990-1A	171842	171832	0.09	0.09	0.03	96.01	90.11	47.74
CC.R2	FKDN220495991-1A	163563	163555	0.08	0.08	0.03	97.02	92.12	47.64
CC.R3	FKDN220495992-1A	176654	176642	0.09	0.09	0.03	96.51	91.41	47.85
SI.C.R1	FKDN220495993-1A	172905	172897	0.09	0.09	0.03	95.93	89.99	48.71
SI.C.R2	FKDN220495994-1A	170988	170726	0.09	0.09	0.03	96.4	90.48	48.84
SI.C.R3	FKDN220495995-1A	174009	173946	0.09	0.09	0.03	95.88	89.74	48.37
C.S1	FKDN220495996-1A	151653	151445	0.08	0.08	0.03	95.93	89.99	50.71
C.S2	FKDN220495997-1A	181719	181615	0.09	0.09	0.03	96.44	91.13	50.56
C.S3	FKDN220495998-1A	153300	153125	0.08	0.08	0.03	96.16	90.7	51.1
SI.S1	FKDN220495999-1A	186888	186846	0.09	0.09	0.03	96.72	92.03	53.89
SI.S2	FKDN220496000-1A	163863	163797	0.08	0.08	0.03	96.28	91	50.71
SI.S3	FKDN220496001-1A	173805	173707	0.09	0.09	0.03	95.69	89.9	52.97
CC.S1	FKDN220496002-1A	165845	165793	0.08	0.08	0.03	96.33	90.86	52.34

CC.S2	FKDN220496003-1A	117731	117694	0.06	0.06	0.03	96.85	91.89	52.16
CC.S3	FKDN220496004-1A	152210	151983	0.08	0.08	0.03	96.5	91.23	52.43
SI.C.S1	FKDN220496005-1A	170370	170217	0.09	0.09	0.03	96.41	91.42	53.19
SI.C.S2	FKDN220496006-1A	154679	154617	0.08	0.08	0.03	95.55	89.7	53.57
SI.C.S3	FKDN220496007-1A	154763	154675	0.08	0.08	0.03	95.26	89.32	54.19

Table S5: Denoising, merging and chimera check for 16S leaf, root and soil samples

sample-id	input	filtered	percentage of input passed filter	denoised	merged	percentage of input merged	non-chimeric	percentage of input non-chimeric
**CD.L1**	154452	137627	89.11	134811	126207	81.71	118117	76.47
**CD.L2**	158647	139985	88.24	139485	138371	87.22	121931	76.86
**CD.L3**	156456	150123	95.95	149627	147773	94.45	140657	89.9
**CD.R1**	153410	132116	86.12	128666	101248	66	93240	60.78
**CD.R2**	154377	130178	84.32	127010	99209	64.26	90135	58.39
**CD.R3**	155120	129795	83.67	125357	99622	64.22	88409	56.99
**CD.S1**	182592	171096	93.7	162696	144388	79.08	112367	61.54
**CD.S2**	171418	160544	93.66	151884	133485	77.87	105520	61.56
**CD.S3**	152266	142472	93.57	135372	119932	78.76	93809	61.61
**CT.L1**	156457	138899	88.78	135548	124663	79.68	116756	74.62
**CT.L2**	153579	136990	89.2	134817	128596	83.73	122869	80
**CT.L3**	153829	137311	89.26	134531	125768	81.76	118888	77.29
**CT.R1**	154082	137468	89.22	130972	102981	66.84	77122	50.05
**CT.R2**	152962	130849	85.54	125031	98292	64.26	88357	57.76
**CT.R3**	156725	133253	85.02	129606	99080	63.22	90098	57.49
**CT.S1**	138526	129132	93.22	118140	95244	68.76	70456	50.86
**CT.S2**	145163	135936	93.64	126189	104608	72.06	77449	53.35
**CT.S3**	166809	156627	93.9	145033	119915	71.89	85608	51.32
**SI+CD.L1**	155824	137835	88.46	135958	130572	83.79	126542	81.21
**SI+CD.L2**	152418	134691	88.37	132224	124898	81.94	116693	76.56
**SI+CD.L3**	155696	140305	90.11	138209	131837	84.68	126517	81.26
**SI+CD.R1**	154769	135603	87.62	133300	108091	69.84	103936	67.16
**SI+CD.R2**	165603	142323	85.94	138631	106082	64.06	96605	58.34
**SI+CD.R3**	154696	131833	85.22	128721	91502	59.15	84543	54.65

**SI+CD.S1**	117442	109797	93.49	103542	90213	76.81	74003	63.01
**SI+CD.S2**	150072	139704	93.09	133373	120542	80.32	98240	65.46
**SI+CD.S3**	145693	135495	93	129342	116433	79.92	93016	63.84
**SI-L1**	154236	137413	89.09	134138	123987	80.39	117611	76.25
**SI.L2**	152175	134545	88.41	132060	124836	82.03	117815	77.42
**SI.L3**	154293	133190	86.32	130831	123384	79.97	114821	74.42
**SI.R1**	155482	132470	85.2	129985	106279	68.35	101249	65.12
**SI.R2**	155644	133363	85.68	129975	99178	63.72	90745	58.3
**SI.R3**	153801	131180	85.29	126525	100459	65.32	91996	59.81
**SI.S1**	189027	178031	94.18	166926	141178	74.69	97243	51.44
**SI.S2**	168206	158209	94.06	150222	124942	74.28	80980	48.14
**SI.S3**	165835	155318	93.66	147181	127190	76.7	97807	58.98

Table S6: Denoising, merging and chimera check for ITS leaf, root and soil samples

sample-id	input	filtered	percentage of input passed filter	denoised	merged	percentage of input merged	non-chimeric	percentage of input non-chimeric
**CD.L1**	152569	132151	86.62	131600	130752	85.7	129470	84.86
**CD.L2**	181059	158271	87.41	157755	156673	86.53	153843	84.97
**CD.L3**	185614	162345	87.46	161340	160238	86.33	158401	85.34
**CD.R1**	171842	138599	80.65	138307	137314	79.91	127037	73.93
**CD.R2**	163563	141266	86.37	140796	139456	85.26	127766	78.11
**CD.R3**	176654	145738	82.5	145409	144189	81.62	131572	74.48
**CD.S1**	165845	132442	79.86	131833	130182	78.5	123946	74.74
**CD.S2**	117731	99663	84.65	99182	96880	82.29	95432	81.06
**CD.S3**	152210	123982	81.45	122466	120311	79.04	116457	76.51
**CT.L1**	149694	117126	78.24	115794	112675	75.27	100694	67.27
**CT.L2**	181493	146797	80.88	145852	144255	79.48	143049	78.82
**CT.L3**	167711	137900	82.22	136887	134812	80.38	130721	77.94
**CT.R1**	185878	144239	77.6	143714	142474	76.65	135953	73.14
**CT.R2**	189628	150196	79.21	148875	144633	76.27	132027	69.62
**CT.R3**	189442	146578	77.37	146176	144897	76.49	138268	72.99
**CT.S1**	151653	117543	77.51	115326	111975	73.84	108409	71.48
**CT.S2**	181719	145099	79.85	143757	141382	77.8	135687	74.67
**CT.S3**	153300	119460	77.93	117732	114939	74.98	110739	72.24
**SI+CD.L1**	186225	148428	79.7	147799	146242	78.53	145661	78.22
**SI+CD.L2**	172046	145263	84.43	144857	143966	83.68	142818	83.01
**SI+CD.L3**	159681	126539	79.24	125852	124882	78.21	124077	77.7
**SI+CD.R1**	172905	136985	79.23	136354	134712	77.91	124908	72.24
**SI+CD.R2**	170988	144326	84.41	143902	143015	83.64	138436	80.96
**SI+CD.R3**	174009	137527	79.03	137142	135790	78.04	132139	75.94

**SI+CD.S1**	170370	137545	80.73	136725	134452	78.92	130022	76.32
**SI+CD.S2**	154679	117081	75.69	116527	114342	73.92	113318	73.26
**SI+CD.S3**	154763	115054	74.34	114424	113353	73.24	112617	72.77
**SI-L1**	187822	160742	85.58	160168	158814	84.56	156346	83.24
**SI.L2**	188682	146677	77.74	145951	144563	76.62	141481	74.98
**SI.L3**	181461	153623	84.66	152868	151488	83.48	148334	81.74
**SI.R1**	156037	131367	84.19	130825	129537	83.02	125050	80.14
**SI.R2**	106149	84219	79.34	83347	81456	76.74	74019	69.73
**SI.R3**	177718	138833	78.12	138124	136230	76.66	128824	72.49
**SI.S1**	186888	153147	81.95	152563	150483	80.52	148166	79.28
**SI.S2**	163863	127529	77.83	126798	124097	75.73	119952	73.2
**SI.S3**	173805	139762	80.41	137248	133662	76.9	132216	76.07

Table S7. Two-way ANOVA multiple comparisons of the Shannon diversity data set

Table Analyzed	ASV-phylum 16S complete				
Two-way ANOVA	Ordinary				
Alpha	0.05				
Source of Variation	% of total variation	P value	P value summary	Significant?	
Interaction	18.41	<0.0001	****	Yes	
plant parts and treatments	62.35	<0.0001	****	Yes	
phyla	1.685	<0.0001	****	Yes	
ANOVA table	SS (Type III)	DF	MS	F (DFn, DFd)	P value
Interaction	6469984158	550	11763608	F (550, 1071) = 9.353	P<0.0001
plant parts and treatments	21912153466	50	438243069	F (50, 1071) = 348.4	P<0.0001
phyla	592155506	11	53832319	F (11, 1071) = 42.80	P<0.0001
Residual	1347049076	1071	1257749		
Data summary					
Number of columns (phyla)	12				
Number of rows (plant parts and treatments)	51				
Number of values	1683				
Table Analyzed	ASV-phylum ITS complete				
Two-way ANOVA	Ordinary				

Alpha	0.05				
Source of Variation	% of total variation	P value	P value summary	Significant?	
Interaction	27.25	<0.0001	****	Yes	
Plant Parts	67.16	<0.0001	****	Yes	
Phyla	0.07574	0.9664	ns	No	
ANOVA table	SS (Type III)	DF	MS	F (DFn, DFd)	P value
Interaction	72153697360	132	546618919	F (132, 299) = 11.12	P<0.0001
Plant Parts	1.77843E+11	12	1.482E+10	F (12, 299) = 301.6	P<0.0001
Phyla	200586187	11	18235108	F (11, 299) = 0.3710	P=0.9664
Residual	14694887497	299	49146781		
Data summary					
Number of columns (Phyla)	12				
Number of rows (Plant Parts)	13				
Number of values	455				

Table S8. Shannon vector values 16S

Sample ID	Organ	Treatments	shannon_entropy	Avg
CT.L1	Shoot*	Control	7.874172971	7.67393
CT.L3		Control	7.473687862	
CT.R1	Root	Control	8.216669276	8.154269
CT.R2		Control	8.54378148	
CT.R3		Control	7.702355691	
CT.S1	Soil	Control	10.56584137	10.45206
CT.S2		Control	10.39029847	
CT.S3		Control	10.40002712	
SI-L1	Shoot	Silicon	7.99799136	7.72984
SI.L2		Silicon	7.736331023	
SI.L3		Silicon	7.45519866	
SI.R1	Root	Silicon	8.025310742	7.584408
SI.R2		Silicon	6.793070733	
SI.R3		Silicon	7.9348435	
SI.S1	Soil	Silicon	10.47844379	9.89743
SI.S2		Silicon	9.563255201	
SI.S3		Silicon	9.650591009	
CD.L1	Shoot**	Cold	7.520351799	7.520352
CD.R1	Root	Cold	8.307954963	8.092962
CD.R2		Cold	7.724004215	
CD.R3		Cold	8.246927652	
CD.S1	Soil	Cold	9.594327145	9.528606
CD.S2		Cold	9.554424208	
CD.S3		Cold	9.437065634	
SI+CD.L1	Shoot	Silicon+cold	7.471342787	7.652478
SI+CD.L2		Silicon+cold	7.750958518	
SI+CD.L3		Silicon+cold	7.735132815	

SI+CD.R1	Root	Silicon+cold	7.992858012	8.172191
SI+CD.R2		Silicon+cold	8.27517874	
SI+CD.R3		Silicon+cold	8.248535464	
SI+CD.S1	Soil	Silicon+cold	9.798214727	9.746435
SI+CD.S2		Silicon+cold	9.791476931	
SI+CD.S3		Silicon+cold	9.649613746	

*n=2, **n=1

Table S9. Shannon vector values ITS

SampleID	Organ	Replicates	Treatments	shannon_entropy	Avg
CT.L1	Leaf	CT.L	Control	5.152316182	4.376002
CT.L2		CT.L	Control	3.855688543	
CT.L3		CT.L	Control	4.120000555	
SI-L1	Leaf	SI.L	Silicon	3.017474648	3.523758
SI.L2		SI.L	Silicon	4.250118947	
SI.L3		SI.L	Silicon	3.303679307	
CD.L1	Leaf	CD.L	Cold	1.171661166	1.843456
CD.L2		CD.L	Cold	3.256235717	
CD.L3		CD.L	Cold	1.102471136	
SI+CD.L1	Leaf	SI+CD.L	Silicon+cold	3.58452589	3.263298
SI+CD.L2		SI+CD.L	Silicon+cold	2.820812532	
SI+CD.L3		SI+CD.L	Silicon+cold	3.384555536	
CT.R1	Root	CT.R	Control	3.570635067	3.888664
CT.R2		CT.R	Control	4.811994966	
CT.R3		CT.R	Control	3.283363195	
SI.R1	Root*	SI.R	Silicon	2.709013107	3.402222
SI.R3		SI.R	Silicon	4.095431737	
CD.R1	Root	CD.R	Cold	2.976943529	3.087747
CD.R2		CD.R	Cold	3.025254509	
CD.R3		CD.R	Cold	3.261042202	
SI+CD.R1	Root	SI+CD.R	Silicon+cold	3.943008677	3.607581
SI+CD.R2		SI+CD.R	Silicon+cold	3.098072519	
SI+CD.R3		SI+CD.R	Silicon+cold	3.781661685	
CT.S1	Soil	CT.S	Control	4.294149713	4.254789

CT.S2		CT.S	Control	4.217544742	
CT.S3		CT.S	Control	4.252674034	
SI.S1	Soil	SI.S	Silicon	3.45490435	3.852949
SI.S2		SI.S	Silicon	3.940369637	
SI.S3		SI.S	Silicon	4.163572243	
CD.S1	Soil	CD.S	Cold	3.735389612	3.778902
CD.S2		CD.S	Cold	3.562881422	
CD.S3		CD.S	Cold	4.038434266	
SI+CD.S1	Soil	SI+CD.S	Silicon+cold	4.047055775	3.792035
SI+CD.S2		SI+CD.S	Silicon+cold	3.785628356	
SI+CD.S3		SI+CD.S	Silicon+cold	3.543420858	

*n=2

Table S10. Permutative multivariate analysis of variance (PERMANOVA) of bacterial and fungal microbiomes across the treatments.

**Bacterial**					
**cold vs control**					
	Df	SumOfSqs	R2	F	Pr(>F)
Treatments	1	0.34128227	0.0667135	0.92927034	0.41
Residual	13	4.77435829	0.9332865	NA	NA
Total	14	5.11564056	1	NA	NA
**Si vs control**					
	Df	SumOfSqs	R2	F	Pr(>F)
Treatments	2	0.5927669	0.0696421	0.86083444	0.53
Residual	23	7.91885058	0.9303579	NA	NA
Total	25	8.51161748	1	NA	NA
**Fungal**					
	**cold vs control**				
	Df	SumOfSqs	R2	F	Pr(>F)
Treatments	1	0.5	0.05882353	1	1
Residual	16	8	0.94117647	NA	NA
Total	17	8.5	1	NA	NA
**Si vs control**					
	Df	SumOfSqs	R2	F	Pr(>F)
Treatments	2	1	0.08	1	1
Residual	23	11.5	0.92	NA	NA
Total	25	12.5	1	NA	NA

Table S11. Total ASV counts

ASV-Phylum-16S						ASV-Phylum-ITS					
	CT	CD	Si	Si+Cd	Total		CT	CD	Si	Si+Cd	Total
Shoot	47220	18325	49261	32422	147228	Shoot	311682	346611	325266	361985	1345544
Root	226380	157008	168543	114583	666514	Root	397953	377703	245553	363231	1384440
Soil	230280	307898	265046	259596	1062820	Soil	336506	321519	379250	332415	1369690
Total	503880	483231	482850	406601	1876562	Total	1046141	1045833	950069	1057631	4099674
*n=2, **n=1						*n=2					
ASV-Genus-16S						ASV-Genus-ITS					
	CT	CD	Si	Si+Cd	Total		CT	CD	Si	Si+Cd	Total
Shoot	37462*	14317**	36424	23332	111535	Shoot	285190	345471	314561	338051	1283273
Root	185606	126535	141419	90694	544254	Root	205543	321765	204198*	326110	1057616
Soil	199805	256216	225725	216463	898209	Soil	270530	281504	317201	274147	1143382
Total	422873	397068	403568	330489	1553998	Total	761263	948740	835960	938308	3484271
*n=2, **n=1						*n=2					

Table S12. Phylum ASVs for bacterial microbiome

	CD.L1	CD.R1	CD.R2	CD.R3	CD.S1	CD.S2	CD.S3	CT.L1	CT.L3	CT.R1	CT.R2	CT.R3	CT.S1	CT.S2	CT.S3
Chloroflexi	101	166	181	441	2050	1699	1560	154	108	495	467	474	3935	3966	4428
Abditibacteriota	9	74	49	94	74	61	41	19	23	74	111	84	6	16	41
Actinobacteriota	1743	4005	3240	3613	10927	5538	4719	2786	1937	10035	9773	4470	5719	5506	7638
Myxococcota	676	1608	1237	2220	3870	3806	3369	1901	979	5752	4860	4344	2624	2951	3201
Patescibacteria	187	538	493	817	437	370	345	333	158	271	799	1147	337	315	660
Acidobacteriota	252	1174	1236	1954	5776	4075	3508	372	363	1468	1818	1571	9543	8749	9098
Elusimicrobiota	25	42	21	132	267	360	314	22	10	92	54	83	241	259	221
Bacteroidota	802	6415	4149	6285	11612	12542	12292	3813	1113	5946	8660	16427	4503	5990	7791
WPS-2	17	46	68	108	428	185	152	5	5	0	52	52	5	11	37
Marinimicrobia_(SAR406_clade)	0	2	0	0	0	0	0	0	0	0	0	0	0	0	0
FW113	0	0	0	0	0	0	0	0	0	0	4	0	0	0	0
Firmicutes	1346	666	237	486	4417	4034	3826	1424	1367	1656	4248	365	3026	2452	4129
Fusobacteriota	7	15	0	5	0	7	0	0	0	0	0	2	0	0	0
Proteobacteria	12009	26394	30543	41679	46888	49463	42436	14157	12614	33755	44764	36717	25350	31496	28542
Bdellovibrionota	393	1230	1099	2157	2499	2189	1908	454	332	1871	1926	1518	651	653	1221
Fibrobacterota	0	49	64	58	163	210	119	34	30	128	216	107	134	103	190
Verrucomicrobiota	557	2434	1822	3991	4217	3020	3275	1308	756	7017	3735	4914	2075	2706	3187
Dependentiae	14	82	114	82	86	116	89	12	35	20	80	66	16	36	46
Armatimonadota	3	82	51	85	1012	940	898	43	31	89	120	149	644	633	838
Deinococcota	0	0	0	0	32	28	18	0	0	0	0	0	109	147	6
Dadabacteria	0	0	0	0	11	0	8	0	0	0	0	0	25	66	13
Campilobacterota	0	0	0	0	0	0	0	0	0	0	0	0	2	0	0
Desulfobacterota	0	20	25	9	192	161	147	3	0	11	14	21	721	586	574

SAR324_clade(Marine_group_B)	3	0	4	0	2	10	9	6	0	7	0	3	10	21	18
NB1-j	3	0	2	0	105	94	74	0	0	0	2	0	346	329	244
Deferrisomatota	0	0	0	0	14	5	0	0	0	0	0	0	60	36	41
Methylomirabilota	0	0	0	0	144	213	174	0	0	0	0	0	706	618	792
Synergistota	0	0	0	0	3	0	4	0	0	0	0	0	56	0	0
DTB120	0	0	0	0	2	0	0	0	0	0	0	0	0	0	0
GAL15	0	0	0	0	10	0	12	0	0	0	0	0	37	20	17
Spirochaetota	0	0	0	0	6	12	0	0	0	0	0	5	56	2	0
Sumerlaeota	0	8	6	30	154	213	166	0	0	0	20	20	36	50	54
Latescibacterota	0	0	0	3	121	45	37	0	0	0	0	0	381	336	288
Hydrogenedentes	0	0	0	0	6	7	11	0	0	0	0	0	19	60	26
PAUC34f	0	0	0	0	0	0	0	0	0	0	0	0	0	2	0
Zixibacteria	0	4	0	0	0	12	0	0	0	0	0	0	25	11	29
Planctomycetota	56	422	333	787	10908	10313	8844	112	64	111	836	889	3483	3930	4027
Cyanobacteria	3	44	34	44	566	497	480	14	7	32	44	37	153	177	290
WS4	0	0	0	0	0	0	0	0	0	0	0	0	3	0	0
WS2	0	0	0	0	4	4	6	0	0	0	0	0	21	11	10
RCP2-54	0	0	0	0	44	55	45	0	0	0	0	0	102	106	67
MBNT15	0	0	0	0	31	10	3	0	0	0	0	0	122	104	149
Entotheonellaeota	0	0	0	0	101	32	43	0	0	0	0	0	297	224	253
Gemmatimonadota	114	404	297	699	3469	3733	3710	133	178	512	567	396	3573	3285	5257
Nitrospirota	5	0	0	0	240	135	174	3	2	7	0	0	413	724	605

Table S13. Phylum ASVs for fungal microbiome

	Olpidiomycota	*Ascomycota*	*Basidiomycota*	*Mortierellomycota*	*Kickxellomycota*	*Mucoromycota*	*Glomeromycota*	*Calcarisporiellomycota*	*Aphelidiomycota*	*Zoopagomycota*	*unidentified*	*Chytridiomycota*	*Rozellomycota*
CD.L1	0	1972	193	0	0	0	0	0	0	0	116418	0	207
CD.L2	0	8426	20888	4	0	0	0	0	0	0	55383	0	0
CD.L3	0	2034	267	0	0	0	0	0	0	0	140810	0	144
CD.R1	0	112421	13695	0	0	0	0	0	0	0	167	0	0
CD.R2	0	86222	32948	0	0	4	6	0	0	0	1870	0	0
CD.R3	0	87560	41594	0	0	4	0	0	0	0	1312	5	0
CD.S1	0	86575	10622	6	0	544	0	0	0	0	22478	39	0
CD.S2	0	65909	4107	6	0	275	0	0	0	0	21024	0	0
CD.S3	0	71737	7888	14	0	538	0	0	0	0	29947	22	9
CT.L1	0	54308	13263	27	0	21	2	0	0	0	7586	0	28
CT.L2	0	36241	12808	1114	0	4	0	0	0	0	72509	0	0
CT.L3	0	38919	15916	12	0	0	0	0	0	0	59230	4	0
CT.R1	0	101538	28838	0	0	0	0	0	0	0	4294	0	0
CT.R2	0	110401	13315	20	0	45	19	0	0	0	2908	0	7

CT.R3	0	114819	19192	6	0	2	0	0	0	0	2776	0	10
CT.S1	0	80949	2562	256	0	387	0	0	0	0	18367	38	883
CT.S2	0	100218	7199	58	0	421	0	0	2	0	19857	0	702
CT.S3	0	85048	5282	58	0	231	0	0	0	0	13712	4	601
SI+CD.L1	0	44409	21549	0	0	0	0	0	0	0	65763	0	0
SI+CD.L2	0	26695	6412	0	0	0	0	0	0	0	88216	0	0
SI+CD.L3	0	35002	10330	0	0	0	0	0	0	0	63736	17	0
SI+CD.R1	0	101298	16684	0	0	7	0	0	0	0	3109	2	0
SI+CD.R2	0	111415	18617	0	0	0	0	0	0	0	1565	0	0
SI+CD.R3	0	91613	17478	0	0	126	0	0	0	0	1428	0	8
SI+CD.S1	0	87083	748	0	0	1248	0	0	0	0	32238	0	0
SI+CD.S2	0	73687	714	0	0	489	0	0	0	0	29012	5	0
SI+CD.S3	0	66103	1096	0	0	331	0	0	0	0	39747	30	0
SI-L1	0	23389	16179	0	0	0	5	0	0	0	95110	3	0
SI.L2	0	51107	12268	5	0	36	0	0	0	0	39034	6	0
SI.L3	0	19001	17208	0	0	0	0	0	0	0	52157	0	0

SI.R1	0	109633	10867	620	0	0	8	0	0	0	2860	0	0
SI.R3	12	94087	20120	0	0	13	0	0	0	0	7447	15	8
SI.S1	0	74735	3773	0	0	571	0	0	5	0	63523	61	260
SI.S2	0	92884	9474	20	0	456	0	0	0	0	11908	35	0
SI.S3	0	51466	4398	301	8	366	4	3	0	3	65195	13	46

Table S14. ANCOM_BC data output, bacterial Phyla

	taxon	lfc_(Intercept)	se_(Intercept)	p_(Intercept)	q_(Intercept)	diff_(Intercept)	diff_TreatmentsControl	diff_TreatmentsSilicon	diff_TreatmentsSilicon+cold
3	Family:Acidobacteriaceae_(Subgroup_1)	0.782615823	0.214531929	0.000264274	0.02923234	TRUE	TRUE	FALSE	FALSE
31	Genus:uncultured_2	1.208540416	0.304688212	7.29E-05	0.012655294	TRUE	TRUE	TRUE	FALSE
37	Genus:Nocardioides	0.440248329	0.151442501	0.003648748	0.194787035	FALSE	TRUE	FALSE	FALSE
47	Genus:Actinospica	0.735322099	0.325004504	0.023666641	0.403080586	FALSE	TRUE	FALSE	FALSE
70	Genus:Curtobacterium	-0.588720008	0.185684465	0.001521559	0.095438083	FALSE	FALSE	FALSE	TRUE
72	Genus:Cellulomonas	-0.450782471	0.332185441	0.17477468	0.95779694	FALSE	TRUE	FALSE	FALSE
77	Family:Streptosporangiaceae	-1.214573556	0.49201661	0.013565711	0.331302801	FALSE	TRUE	FALSE	FALSE
82	Genus:Paenarthrobacter	0.400839516	0.151015971	0.007947653	0.262698623	FALSE	TRUE	FALSE	FALSE
168	Genus:uncultured_21	-0.588535616	0.303839454	0.052745909	0.600092804	FALSE	TRUE	FALSE	FALSE
183	Genus:Acidipila	0.626271622	0.163542996	0.000128456	0.017829669	TRUE	TRUE	FALSE	FALSE
189	Genus:Granulicella	1.33740256	0.27236536	9.09E-07	0.000315511	TRUE	TRUE	FALSE	TRUE
233	Genus:Mucilaginibacter	1.281918718	0.400160043	0.001357609	0.094218097	FALSE	FALSE	FALSE	TRUE

259	Genus:Bdellovibrio	0.54461364	0.150454794	0.000294851	0.02923234	TRUE	TRUE	FALSE	FALSE
261	Order:Chlamydiales	-0.430080732	0.254645128	0.091230635	0.73621001	FALSE	TRUE	FALSE	FALSE
264	Genus:WPS-2	1.358133922	0.487932758	0.005378501	0.208365695	FALSE	TRUE	FALSE	FALSE
276	Genus:Blautia	0.861233591	0.324476552	0.007949094	0.262698623	FALSE	TRUE	FALSE	FALSE
292	Genus:Ahniella	-0.777492636	0.186428922	3.04E-05	0.007032481	TRUE	TRUE	FALSE	FALSE
397	Genus:Brevundimonas	-0.715947539	0.347890394	0.039593139	0.528416121	FALSE	FALSE	FALSE	TRUE
413	Genus:Fusobacterium	0.889285548	0.25488839	0.000484974	0.042071477	TRUE	TRUE	FALSE	FALSE
424	Genus:Acidisphaera	0.763184413	0.301616046	0.011395929	0.304183652	FALSE	TRUE	TRUE	FALSE
435	Genus:Herpetosiphon	-0.697085351	0.261836464	0.007761032	0.262698623	FALSE	TRUE	FALSE	FALSE
437	Genus:Burkholderia-Caballeronia-Paraburkholderia	0.699227519	0.275984158	0.011290446	0.304183652	FALSE	TRUE	FALSE	FALSE
468	Genus:Bacteriovorax	0.340707744	0.175152882	0.051750665	0.600092804	FALSE	TRUE	FALSE	FALSE
485	Genus:RS25G	0.924196188	0.186578071	7.29E-07	0.000315511	TRUE	TRUE	TRUE	TRUE
488	Genus:FukuN18_freshwater_group	0.838970556	0.354746007	0.018030586	0.338195311	FALSE	TRUE	FALSE	FALSE

533	Family:Gallionellaceae	-0.453454525	0.204053785	0.026267739	0.424157198	FALSE	TRUE	FALSE	FALSE
557	Genus:Holophaga	0.435260987	0.240130631	0.069893406	0.646746986	FALSE	TRUE	FALSE	FALSE
575	Genus:Caproiciproducens	-0.364829181	0.153591348	0.017533563	0.338008125	FALSE	FALSE	FALSE	TRUE
585	Genus:uncultured_98	-0.401635581	0.206272653	0.051521528	0.600092804	FALSE	FALSE	FALSE	TRUE
620	Genus:CCM11a	-0.454773104	0.144515549	0.001650226	0.095438083	FALSE	TRUE	FALSE	FALSE
642	Genus:Pelosinus	-0.459660416	0.162548982	0.004686511	0.20327742	FALSE	TRUE	FALSE	FALSE
644	Genus:Candidatus_Entotheonella	-0.432953329	0.169802071	0.010779928	0.304183652	FALSE	TRUE	FALSE	FALSE
671	Genus:Agromyces	-0.397896823	0.180336101	0.027354745	0.424157198	FALSE	TRUE	FALSE	FALSE
682	Genus:uncultured_115	0.569774782	0.23304676	0.014489445	0.331302801	FALSE	TRUE	FALSE	FALSE
690	Genus:Phyllobacterium	-0.432681166	0.233983383	0.064429498	0.63527264	FALSE	TRUE	FALSE	FALSE
